# Photodynamic therapy, priming and optical imaging: Potential co-conspirators in treatment design and optimization — a Thomas Dougherty Award for Excellence in PDT paper

**DOI:** 10.1142/s1088424620300098

**Published:** 2020

**Authors:** Pushpamali De Silva, Mohammad A. Saad, Hanna C. Thomsen, Shazia Bano, Shoaib Ashraf, Tayyaba Hasan

**Affiliations:** a Wellman Center for Photomedicine, Massachusetts General Hospital and Harvard Medical School, Boston, MA 02114, USA; b Division of Health Sciences and Technology, Harvard University and Massachusetts Institute of Technology, Cambridge, MA 02139, USA

**Keywords:** photodynamic therapy, photodynamic priming, combination therapies, immunogenic cell death, photoimmunoconjugates, optical imaging, antimicrobial PDT

## Abstract

Photodynamic therapy is a photochemistry-based approach, approved for the treatment of several malignant and non-malignant pathologies. It relies on the use of a non-toxic, light activatable chemical, photosensitizer, which preferentially accumulates in tissues/cells and, upon irradiation with the appropriate wavelength of light, confers cytotoxicity by generation of reactive molecular species. The preferential accumulation however is not universal and, depending on the anatomical site, the ratio of tumor to normal tissue may be reversed in favor of normal tissue. Under such circumstances, control of the volume of light illumination provides a second handle of selectivity. Singlet oxygen is the putative favorite reactive molecular species although other entities such as nitric oxide have been credibly implicated. Typically, most photosensitizers in current clinical use have a finite quantum yield of fluorescence which is exploited for surgery guidance and can also be incorporated for monitoring and treatment design. In addition, the photodynamic process alters the cellular, stromal, and/or vascular microenvironment transiently in a process termed photodynamic priming, making it more receptive to subsequent additional therapies including chemo- and immunotherapy. Thus, photodynamic priming may be considered as an enabling technology for the more commonly used frontline treatments. Recently, there has been an increase in the exploitation of the theranostic potential of photodynamic therapy in different preclinical and clinical settings with the use of new photosensitizer formulations and combinatorial therapeutic options. The emergence of nanomedicine has further added to the repertoire of photodynamic therapy’s potential and the convergence and co-evolution of these two exciting tools is expected to push the barriers of smart therapies, where such optical approaches might have a special niche. This review provides a perspective on current status of photodynamic therapy in anti-cancer and anti-microbial therapies and it suggests how evolving technologies combined with photochemically-initiated molecular processes may be exploited to become co-conspirators in optimization of treatment outcomes. We also project, at least for the short term, the direction that this modality may be taking in the near future.

## INTRODUCTION

**Fig. 1.**
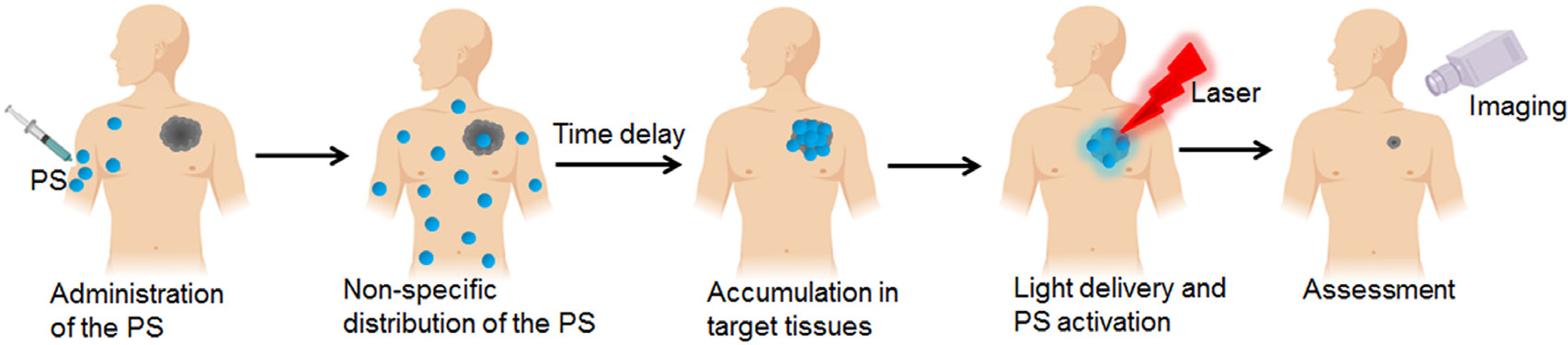
Photodynamic therapy as a single modality for therapy and imaging. Photosensitizer (PS) is administered systemically, following which it preferentially localizes at the desired site. The time delay following PS administration and its subsequent irradiation is referred to as the drug-light-interval. Irradiation of the PS results in reactive molecular species generation and fluorescence emission, which could be used for inducing cytotoxicity and imaging, respectively.

Photodynamic therapy (PDT) is a United States Food and Drug Administration (FDA) and European Medicines Agency (EMEA) approved modality for the treatment of a number of cancer and non-cancer pathologies [1, 2]. While this review discusses the mechanistic aspects of PDT and its general applications, the reader is referred to excellent recent reviews updating the clinical status of PDT world-wide [3–5]. PDT involves the administration of a light activatable non-toxic chemical, which upon irradiation with an appropriate wavelength of light leads to the production of reactive molecular species (RMS), to induce cytotoxicity at target sites. A major attribute of PDT is the dual selectivity that it confers due to the preferential accumulation of the photosensitizing agent in the desired tissues/cells and the confined volume of irradiation so that collateral damage is minimized (Fig. 1). The phenomenon of preferential accumulation is not entirely understood, and neither is it universal for all sensitizers and site of action. However, depending on the anatomical site, the ratio of tumor to normal tissue may be reversed in favor of normal tissue. Under such circumstances, control of the volume of light illumination provides a second handle of selectivity. As with chemotherapeutics, selectivity of untargeted photosensitizers (PSs) is typically attributed to factors of enhanced permeability and retention (EPR) effect, the dysfunctional leaky nature of blood vessels in tumors and some other pathologies, and perhaps lack of efficient lymphatic drainage [6]. The typical routes of administration of the photosensitizing agent are intravenous, oral, or topical. While intravenous administration of PSs is most common for internal disease sites, oral and topical application is generally preferred for the treatment of localized lesions such as leukoplakia [7], oral cancers [8], dentistry [9] and several dermatological conditions such as actinic keratosis, basal cell carcinoma and bowen’s disease [10]. While, aminolevulinic acid (ALA) and its derivatives, including methyl aminolaevulinate (MAL) and the nanoemulsion BF-200 ALA, are the most popular photosensitizers for topical and oral administration, development of other PSs including hypericin and silicon phthalocyanine have also been reported [11, 12]. A unique feature of photodynamic activation is the potential for use of a single molecular entity for both therapy and imaging [13–20], opening up possibilities for real-time treatment guidance and monitoring. This is a desirable attribute of the technology as there is an increasing use of image-guided therapies for diagnosis and therapeutic interventions. Magnetic resonance imaging (MRI), computed tomography (CT), endoscopy, and X-ray fluoroscopy are already integrated into medical systems and often provide information for prognosis or therapy-guidance. Image-guided therapies can be minimally invasive and more localized, making them a popular choice in the clinic [21, 22]. Although this review will not focus on the significant advances made in optical imaging with PDT agents, we incorporate some salient examples where such an approach is used in treatment guidance and monitoring.

The therapeutic potential of light has been known for thousands of years [13]. However, Raab’s work in the 1900s with *Paramecia* incubated with acridine orange and accidental exposure to sunlight, resulting in cytotoxicity to the organism, was possibly one of the key early observations that led to the current version of PDT applications [23, 24]. The discovery of hematoporphyrin derivative (HpD), a crude mixture of porphyrins, by Schwartz and Lipson [13, 25–27], developed further by Tom Dougherty, showing preferential accumulation in tumors of some components of this mixture, formed the basis of the FDA approved, relatively purified version of HpD called Photofrin (Pf). Currently, Pf is in clinical use for various oncological applications, including the management of lung, skin metastasis, bladder malignancies, and esophageal cancers [2, 13, 28–30]. These initial approvals have been followed by the development of several new PDT agents which have shown potential in both oncologic and non-oncologic applications [31–33], and many have been approved by regulatory authorities. This review is by no means meant to be comprehensive regarding PDT and discusses limited aspects of the subject.

## MECHANISMS UNDERLYING PDT

As discussed above, PDT involves photochemical and photophysical processes, which results in the subsequent biological outcomes. In addition to the direct toxicity and death that the PDT process (vascular or cellular) provides, there is an additional sub-lethal photochemistry induced biological effect which primes the cellular, stromal and/or vascular microenvironment for subsequent treatment with other modalities and is referred to as photodynamic priming (PD*P*). An exciting outcome of PD*P* is the sensitization of tumors for enhanced secondary therapies, such as immune-, chemo-therapy, and other inhibitory therapies, including receptor tyrosine kinase inhibition (RTKi) (discussed in a later section) [18, 34–43] making PD*P* an enabler of the more commonly used therapies. The various mechanisms associated with PDT are briefly discussed in this section.

### Photochemistry and photophysics in PDT: A simplistic version

**Fig. 2.**
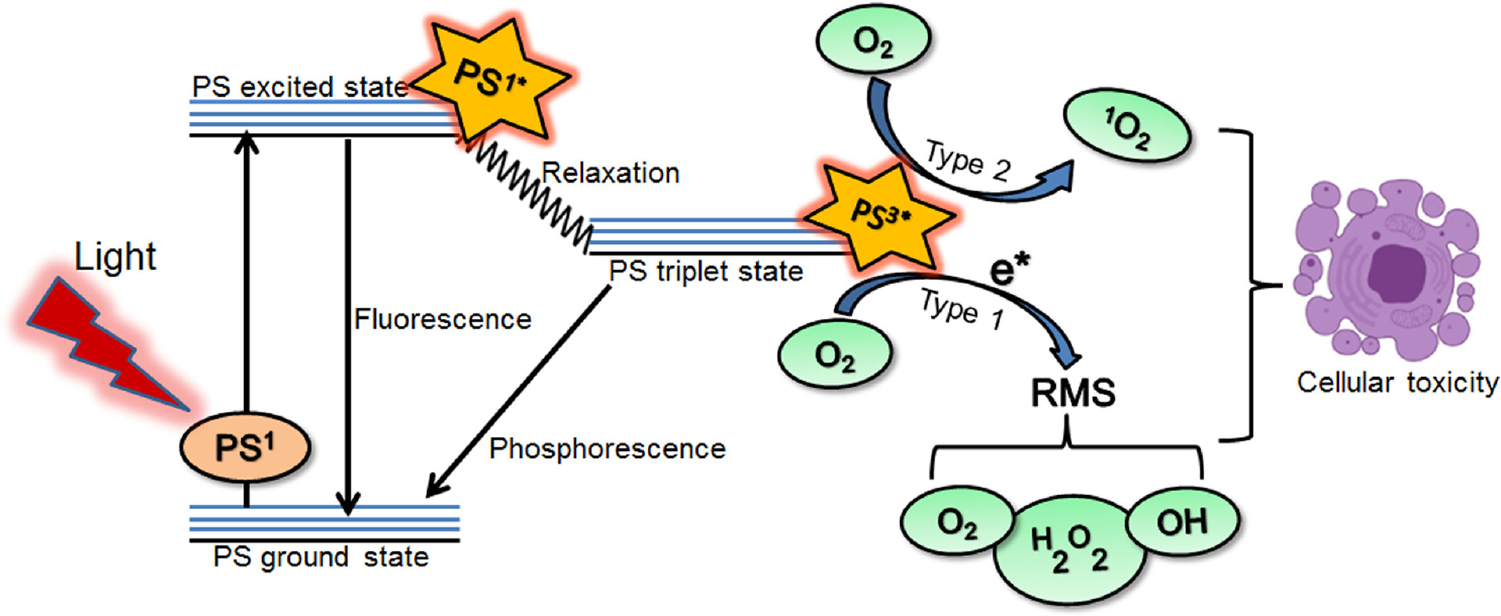
Photochemical and photophysical reactions associated with photodynamic therapy. When the photosensitizer (PS; ground state) absorbs light (photon) at a particular wavelength, it is first excited to singlet state (PS^1*^) and then converted to a more stable triplet state (PS^3*^). This triplet state (PS^3*^) can react with molecular oxygen and other biomolecules through the so-called type 1 and type 2 reactions, creating highly reactive molecular species (RMS) and singlet oxygen (^1^O_2_), both of which can cause cellular toxicity.

In PDT, a nontoxic light-activatable chemical, a PS, is administered either systemically or locally, after which the PS preferentially accumulates in the target tissues/cells [13–18]. Upon irradiation at a particular wavelength, the ground state photosensitizer (PS^1^) is excited to a singlet excited state (PS^1*^) (Fig. 2). From this state, the PS^1*^ can either relax to the ground state through radiative transfer of energy (fluorescence) or undergo intersystem crossing resulting in the formation of a long-lived excited triplet state (PS^3*^). The subsequent energy transfer to ground state triplet oxygen (^3^O_2_) from the PS^3*^ (the so-called type 2 reactions) leads to the formation of toxic singlet oxygen species. However, transfer of electrons (the so-called type 1 reactions) from the PS^3*^ to other biomolecules, including water and oxygen, results in the formation of RMS (Fig. 2). Most of the PSs used are based on a tetra-pyrrole structure, similar to that of protoporphyrin group in hemoglobin [13, 44, 45] with one absorption peak between 600 and 800 nm (red to deep/near infra-red; NIR). This makes them ideal PSs, as lower wavelength light does not penetrate efficiently through the tissue and light at longer wavelengths (above 800 nm) does not have sufficient energy to initiate a photochemical reaction and generate a substantial amount of RMS [13, 44, 45]. In addition, as mentioned above, these PSs have a finite quantum yield of fluorescence, therefore they can be used both as imaging and therapeutic agents [13]. The accumulation of PSs in tumors can be higher than surrounding normal tissues, as discussed earlier, but that is not always the case and is dependent on the site of disease. The sensitizer can be present in higher amounts in specific compartments of target tissue such as the vasculature due to either the structure of the molecule or simply due to temporal considerations [46]. The cellular uptake of PSs can occur either by passive diffusion or by active endocytosis [13].

### Subcellular localization and cell death pathways associated with PDT

Until approximately the early 1990s, a generally accepted mechanism of PDT-based treatment of tumors *in vivo* was believed to be RMS-mediated vascular damage leading to tissue necrosis in the area where light was applied [2, 13, 47]. More recently it has been appreciated that other cell death pathways, depending on PS properties, their intracellular localization, drug light interval (DLI), and PDT dose (PS concentration * light fluence), are involved [48–50]. Different organelles display differences in susceptibility to the generated RMS and hence show variations in the extent of PDT-mediated damage. Formulating PSs to enhance their accumulation in specific intracellular locations, where RMS production can offer potential enhancement in PDT efficacy, has also been attempted [51].

However, there is not a clear consensus of PS’s localization to a particular organelle being more effective for PDT-based treatments. It is known that PSs that target mitochondria are potent cytotoxicity inducers [52, 53] and most of the clinically approved PSs including Foscan [54], Pf [55], and Visudyne [56] are known to partially localize in the mitochondria [57–59]. PDT-induced mitochondrial photodamage, first reported by Kessel *et al.* [57, 60, 61] and confirmed by Oleinick *et al.* [62], has been shown to result in loss of mitochondrial membrane potential. Hence, activation of the cancer cell death mechanisms, either by inhibiting tumor-specific adaptation of the mitochondrial metabolism or by enhancing mitochondrial damage, could, therefore, be another promising therapeutic approach [63]. Also, PSs that target ER and other organelles (such as lysosomes) in the cytoplasm [64] or simultaneously target multiple subcellular organelles [65–67] are effective in PDT-mediated tumor cell death. Benefits of combining PSs for targeted lysosomal photodamage followed by, or simultaneously with, mitochondrial photodamage, have been previously established to enhance PDT efficacy [31, 59, 66, 68–70]. A combination of different PSs which localize to different subcellular organelles have been shown to be effective in inducing cancer cell death by Villanueva *et al.* [71] and others [66, 70, 72]. More recently, a single PS; Benzoporphyrin derivative (BPD) loaded in two different liposomal formulations as free BPD and lipid anchored BPD, was shown to localize in different organelles and significantly enhanced PDT efficacy, in monolayer cell culture, relative to either formulations alone [68]. Building on these results, Rizvi *et al*., reported the enhanced therapeutic benefits of a combined treatment of two liposomal formulations of BPD, clinically approved Visudyne and a lipid-anchored BPD, in a 3D model of ovarian cancer, due to the simultaneous PS localization and photodestruction of mitochondria/endoplasmic reticulum (ER) and lysosomes [65]. In this context, it is worth noting that some PSs have been shown to undergo relocalization to different subcellular sites during the process of irradiation [73–75]. During exposure to low light doses, PSs localized into specific subcellular organelles such as lysosomes or ER can relocate to the cytoplasm in general, and, more specifically, to the nucleus [73–76]. This is attributed to photodynamic permeabilization of the lysosomal or ER membrane, thus allowing small molecules, including the PS to leak out into the cytoplasm. This effect of PS redistribution may result in photoinactivation of enzymes required for programmed cell death pathways such as apoptosis.

**Fig. 3.**
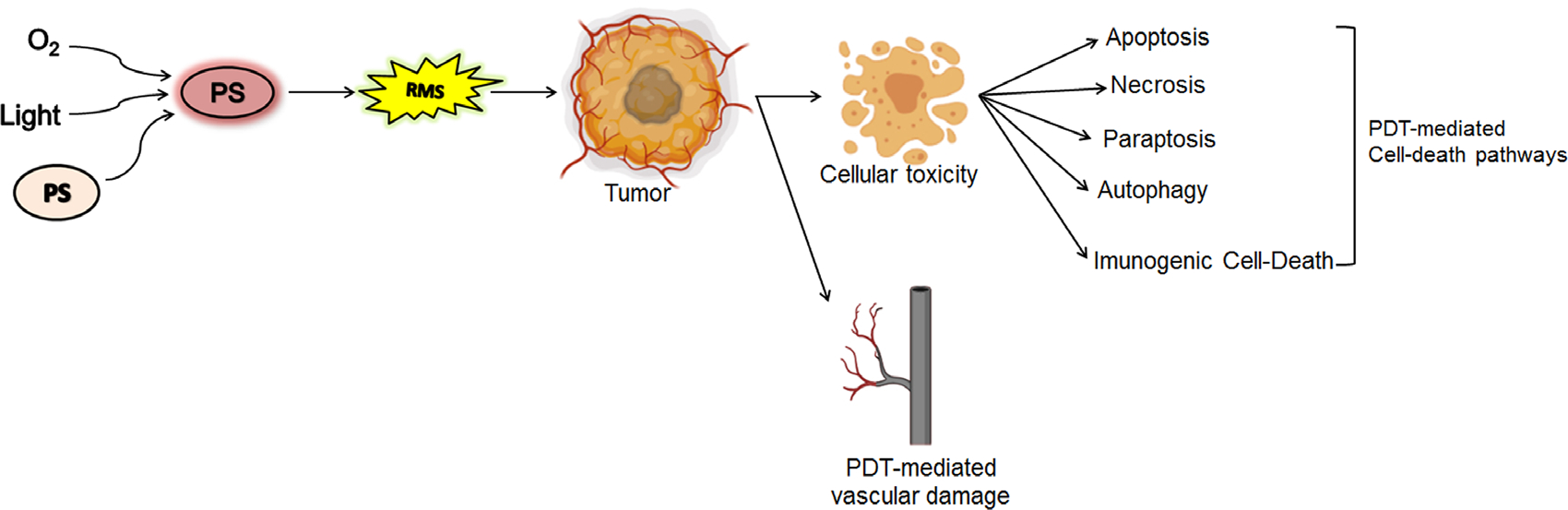
Generation of reactive molecular species (RMS) upon light activation of the photosensitizer (PS) and PDT associated cell death pathways and vascular damage that could occur as a result.

Unlike other treatment regimens, such as chemotherapy, that have known systemic toxicity, cell death pathways induced by PDT may directly impact target cell populations without adverse effects simply because of the dual selectivity. The inter-cellular or intracellular location of PSs is critical in determining the cell death mechanism and thus the cellular response to photodamage [59, 77–80]. These cell death mechanisms include necrosis, apoptosis, autophagy, paraptosis, ferroptosis, pyroptosis, necroptosis and immunogenic cell death [59, 65, 80–83]. The diversity in cellular responses, as observed post-PDT, provides an exceptional opportunity for the exploitation of PDT and the design of complementary combination therapies for cancer and other diseases (Fig. 3). In this section, we briefly describe the different cell death pathways mediated by PDT.

#### Necrosis.

**Fig. 4.**
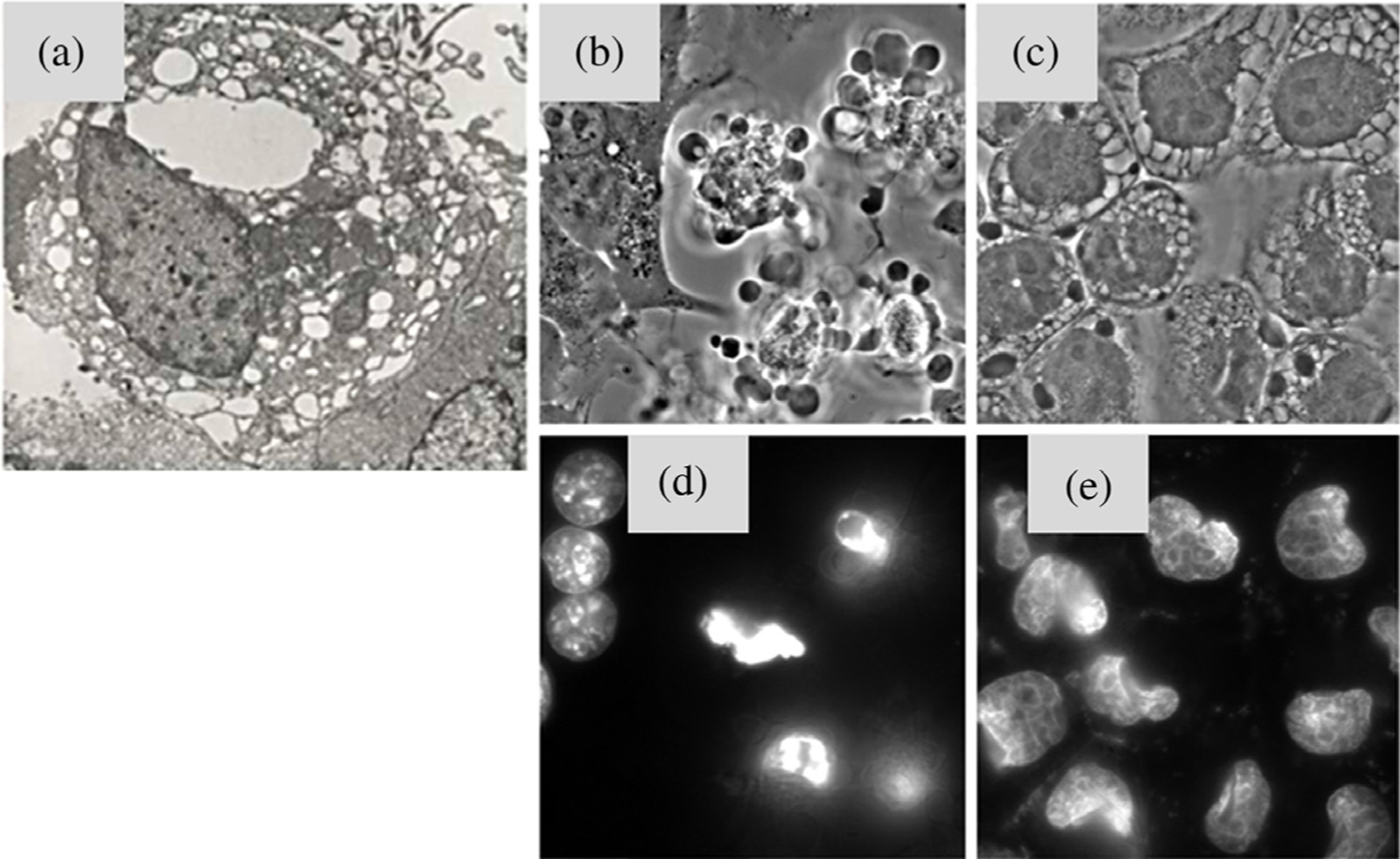
Cellular changes associated with (a) necrosis, (b, d) apoptosis and (c, e) paraptosis. Panels (a, b) and (c) show phase-contrast images of cells undergoing apoptosis and paraptosis (d) Nuclear condensation and fragmentation typical of apoptosis. (e) Nuclear condensation and fragmentation are not observed during paraptosis. The fluorescent label Ho33342 was used to probe nuclear morphology in panels (d) and (e). Figure (a) adapted from Lee *et al.* (2018) [85]) and Figure panels (b–e) adapted from Kessel and Oleinick (2018) [80].

Necrosis is an uncontrollable cell death, which occurs due to physical or chemical cellular damage and is characterized by a pyknotic nucleus, cytoplasmic swelling, and progressive disintegration of cytoplasmic membranes (Fig. 4a) [84, 85]. This leads to cellular fragmentation and spillage of cellular contents into the extracellular environment, stimulating an inflammatory response predominantly involving neutrophil infiltration [86]. The early understanding of PDT mediated cell death was dominated by this mode of death, where tissue necrosis due to vascular damage and cell membrane localizing PSs was the leading mechanistic pathway [65, 80, 81, 87]. The factors and parameters that cause cellular necrosis after PDT are not clear, but it has been shown that high dose PDT (either a high PS concentration or a high light fluence or both) tends to cause cell death by necrosis [64, 88]. Importantly, as suggested in early studies, an inflammatory response associated with necrotic cell death is possibly the major cause of immune stimulation and tumor antigen spread, post–PDT [37, 89–91].

#### Apoptosis.

Apoptosis or programmed cell death is characterized by the unique morphological and energy-consuming series of tightly controlled cellular reactions taking place at the sub-cellular level in every normal cell of the body [63, 92, 93]. This process can occur *via* two main initiating routes involving either the activation of death receptors (the extrinsic pathway) or the mitochondrial release of cytochrome c (the intrinsic pathway). Alternatively, generation of tBid through the extrinsic pathway can trigger the intrinsic pathway and amplify the apoptotic response. Ultimately, these pathways converge at the caspase activation step and lead to cellular changes such as poly nucleosomal DNA fragmentation, plasma membrane blebbing, and nuclear shrinking, which could be morphologically and biochemically detected in dying cells (Figs 4b and 4d). [94–96]. It is well-studied that tumor cells can be resistant to apoptosis due to the over-expression of anti-apoptotic machinery (Bcl-2 rheostat) [97, 98] operating inside these cells allowing their survival and metastases. The application of chemotherapeutic agents, radiation, and PDT has been known to trigger apoptosis [80, 87, 98, 99], that can be hindered by the overexpression of anti-apoptotic protein Bcl-2. The unique aspect about PDT is that it can bypass the extrinsic apoptotic pathway and the regulatory effects of these anti-apoptotic proteins leading to a direct release of cytochrome c and activation of caspases, thus making it agnostic to important mechanisms of resistance [49, 100, 101].

The first report of PDT-mediated apoptosis comes from experiments carried out with chloroaluminum phthalocyanine as the PS, in mouse lymphoma cells, which showed dose and time-dependent DNA fragmentation [48]. As mitochondria are decisive regulators of apoptosis and also important for their role in the energy metabolism of a cell, their targeting has been shown to maximize PDT potency [102, 103]. Moreover, PSs that target mitochondria or ER can lead to the destruction of mitochondrial outer membrane-associated Bcl-2 and Bcl-xL and the activation of pro-apoptotic proteins such as Bax and Bak [81, 101, 104, 105]. These events lead to cytochrome c release and subsequent activation of caspase 9 and other executionary caspases, eventually leading to apoptotic reactions evident by the degradation of multiple cellular components, such as proteins and DNA. In contrast, lysosomal photodamage is not as obvious as the mitochondria or ER induced apoptosis process. However, the release of lysosomal proteases cleaves the cytosolic protein, Bid to a truncated form tBid, inducing an apoptotic response [81]. Kessel *et al.* demonstrated that a sequential low dose of lysosomal and mitochondrial PDT could achieve synergistic photokilling *via* enhancement of the proapoptotic signaling pathways [66]. Interestingly, apoptotic cell death induced by photodamage can be quite rapid, resulting in the formation of DNA “ladders” and condensed/fragmented chromatin can be detected within an hour after PDT [106].

#### Paraptosis.

Paraptosis is initiated with the dilation of ER and mitochondria in response to misfolded proteins (Figs 4c and 4e) [86, 107, 108]. This form of cell death can occur due to an exposure to chemotherapeutic agents [109–111] or photodamage [69, 87, 112, 113] leading to the accumulation of multiple vacuoles that gradually fill the cytoplasm. Relatively little is known regarding the molecular basis of paraptosis, however, the underlying mechanism clearly differs from that of apoptosis. Also, there is evidence that a so-called “canonical pathway” involving Mitogen-activated protein kinases (MAPKs) [109, 114] and “non-canonical pathway” implicated in the effect of an anti-tumor agent, Taxol, that promotes tubulin polymerization and blocks the progression of mitosis, which is independent from MAPKs signaling pathway [115], may be involved in PDT-mediated paraptosis. Recent studies have pointed out that the relocation of High Mobility Group Box 1 (HMGB1) from the nucleus to plasma membrane [116] and altered expression of IGF-1R and AIP-1/Alix proteins [111] are potential markers for paraptosis. Nevertheless, this form of cell death may take place when apoptosis is restricted in some situations [112, 117]. The role of PDT mediating paraptosis is not clear, however, several reports show the presence of highly vacuolated cytoplasm, post-PDT, suggesting an ongoing paraptosis process may be due to PDT-mediated crosslinking of ER proteins [69, 80, 81, 112, 118]. A recent study by Kessel *et al*. [113] suggests that photodamage to ER appears to initiate canonical and non-canonical pathways of paraptosis depending on the light dose used.

#### Autophagy.

Autophagy is a cellular process for the degradation and elimination of misfolded proteins and damaged organelles in order to maintain cellular homeostasis [119]. The work in PDT related autophagy has been led by the Kessel group and shown to involve intracellular degradation pathway mediated by double-membrane vesicles called autophagosomes that deliver degraded cytoplasmic components to the lysosome for recycling during stressful conditions. Autophagy-related genes (ATG) play an important role in the formation of autophagosomes and the regulation of autophagic cell death [120]. Increasing evidence suggests that autophagy is involved in both the promotion of tumorigenesis and inhibition of cancer [121, 122]. Autophagy may play a dual role in response to PDT depending on which subcellular compartment is targeted. If PDT is directed against mitochondria or ER, autophagy may provide partial cytoprotection from the effects of photodamage by recycling damaged mitochondria or ER before they can induce apoptosis [123, 124]. This has been further demonstrated by the enhancement of cellular cytotoxicity in mitochondrial photodamage when ATGs (ATG5 and ATG7) were knocked down [80, 81, 123, 125, 126]. The autophagic recycling process involves fusion of autophagic vesicles with lysosomes. Thus, photodamage to lysosomes can interrupt autophagy much more effectively. Experimental data has shown that lysosomal photodamage hinders the low pH environment required for the optimal activation of lysosomal proteases which results in an accumulation of autophagosomes that are not processed further, thereby promoting apoptotic cell death [125, 127].

#### Ferroptosis, pyroptosis and necroptosis.

Apart from the above mentioned well-described cell death pathways, there have been reports of other programmed cell death mechanisms, occurring independent of apoptosis, that were discovered recently and have been associated with PDT. However, it is still not clear that these cell death pathways are induced as a direct effect of the PDT process, as suggested in a limited number of studies, discussed briefly in this section.

Ferroptosis, a non-apoptotic iron dependent form of cell death, is usually accompanied by a large amount of iron accumulation and lipid peroxidation during the cell death process [128–130]. Ferroptosis-inducing factors can directly or indirectly affect glutathione biosynthesis or the glutathione-dependent antioxidant enzyme glutathione peroxidase 4 (GPX4) resulting in a decrease in antioxidant capacity and accumulation of lipid-RMS (singlet oxygen) in cells, ultimately leading to oxidative cell death, which is marked by the depletion of plasma membrane polyunsaturated fatty acids. A few reports have highlighted PDT associated ferroptosis [82, 131, 132] in different tumor models and is usually observed when specific PSs are used in the PDT process. Turubanova *et al*., using photosens and photodithazine as PSs, demonstrated inhibition of cell death when ferrostatin-1 (ferroptosis inhibitor) was used in photosens-mediated PDT, highlighting the occurrence of ferroptosis mediated cell death [82]. Another recent study [131] suggests that ferroptosis could enhance PDT efficacy by maintaining a sustainable O_2_ supply (generated through the Fenton reaction). Moreover, the additive effect of lipid-RMS, generated by ferroptosis, and ROS generated by PDT, could potentially enhance cytotoxicity even in tumors where the associated hypoxia is often the cause of low PDT efficacies.

Pyroptosis is an inflammatory form of cell death that has the potential to activate a local or systemic immune response *via* the expression of damage-associated molecular patterns (DAMPs) or pathogen-associated molecular patterns (PAMPs) [133–136]. It is mainly dependent on caspase 1 activation, which is responsible for the maturation of proinflammatory cytokines such as interleukin-1 beta (IL-1β) and IL-18 through inflammasome-dependent pathways. Thus, pyroptosis primarily seems to be associated with inflammatory cells such as macrophages and may be triggered by bacterial or pathogen infections [135]. Cells undergoing pyroptosis may share some similar features with necrotic cell death such as membranous pore formation, cytoplasmic swelling, leakage of cytosolic contents, and also might display DNA fragmentation and nuclear condensation. Increasing evidences suggest that pyroptosis can be chemically induced in cancer cells without any bacterial or viral infection with the potential to affect all stages of tumorigenesis [137–139]. Cell death by pyroptosis has also been implicated in a recent work with PDT in cancer cells [140], expanding exploratory avenues in PDT-mediated cell death mechanisms. However, pyroptosis related cell death features observed in this study were mostly attributed to the sonodynamic activity of the photo/sonosensitizer (curcumin) used.

Necroptosis, another inflammatory type of programmed cell death with some similarities to pyroptosis, mimics features of apoptosis and necrosis, and is identified by membrane permeabilization and cell swelling [141–143]. Membrane pore formation during necroptosis disrupts the integrity of plasma membrane and causes cell death followed by the release of intracellular DAMPs. Necroptosis is often induced by toll-like receptor, death receptor, and interferon signaling [141–143], which could occur as a result of different kinds of physical-chemical stress including anticancer drugs [144, 145], ionizing radiation [146] and PDT [147–149]. Some PSs such as talaporfin sodium [150] and porphyrins [149] have shown to activate necroptosis. Miki *et al*., demonstrated that necroptosis could take place when low concentrations of talaporfin sodium was used during PDT and high concentrations of the same PS caused necrosis in human glioblastoma cells [150]. Further delineating necroptosis pathway associated with PDT may provide the advantage to bypass the resistance to apoptosis observed in cancers cells and induce antitumor immunity.

### Immunogenic cell death

**Fig. 5.**
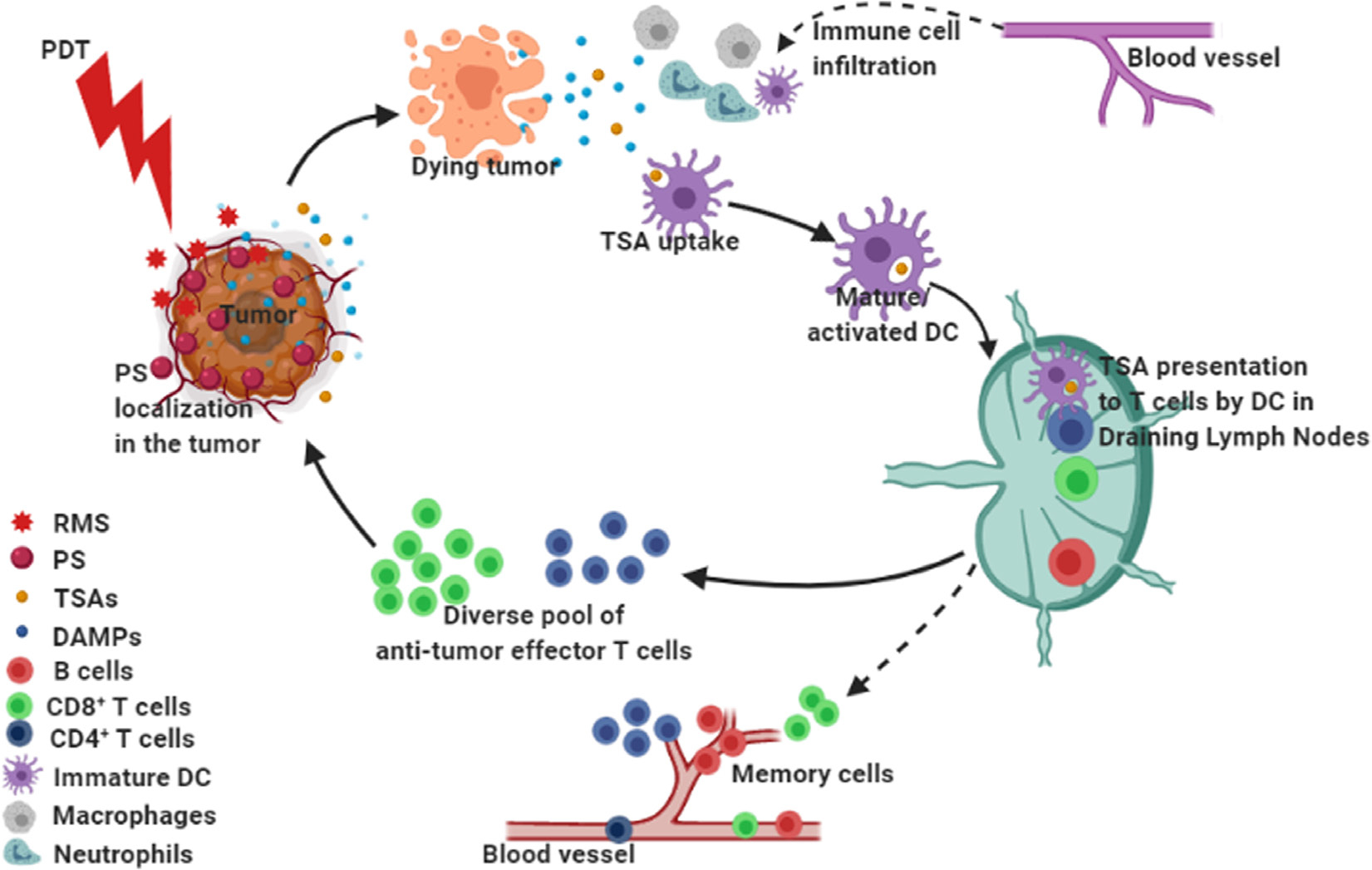
PDT mediated anti-tumor immune processes that may occur in the tumor microenvironment (TME). Irradiation of PS-loaded tumor cells generates RMS that leads to tumor cell death. The photodynamic process results in priming of the TME where the dying cells express or release damage associated molecular patterns (DAMPs). Photodamaged tumor cells and tissue-resident immune cells may release numerous cytokines and chemokines that may induce extravasation of innate immune cells to the tumor site causing local inflammation. Professional antigen presenting cells such as Dendritic cells (DCs) play a key role in bridging the innate immune response with adaptive immunity. Tumor specific antigens (TSAs) captured by immature DCs become activated and migrate to the draining lymph nodes and help to prime naïve T cells (CD4^+^ helper T or CD8^+^ cytotoxic T cells), which differentiate into effector or memory T cell subsets. Anti-tumor effector T cell populations, especially CD8^+^ cytotoxic T cells may migrate to the tumor site in search of the cognate TSAs and kill the tumor cells. We postulate that PDT may enrich a diverse pool of antitumor T cell clones in the TME to kill the tumor and possibly expand long lived memory T and B cells that get into the peripheral circulation to maintain immune surveillance.

Although PDT-mediated tumor destruction primarily occurs *via* various cellular pathways discussed above and vascular shutdown; stimulation of immune responses also plays a vital role in maintaining long-term tumor control. Korbelik and colleagues led the way on PDT induced immunological effects [151–156], and currently this is a very active topic within the field [65]. PDT leads to a severe insult on the tumor and its microenvironment and results in the initiation of a wave of proinflammatory processes [157, 158] which primes the organism for eliciting direct or indirect immunological effects. Early evidence of the release of proinflammatory cytokines, post-PDT, came from the Henderson lab and others [65, 154, 159–161]. There is also release of DAMPs (Fig. 5), post-PDT, which results in a marked increase in the immunogenicity of dying cancer cells leading to the infiltration of host innate immune cells, such as dendritic cells (DCs) or macrophages, which carry out the removal of damaged cells. These innate immune cells can in-turn activate adaptive immune responses by presenting various tumor-specific antigens (TSAs), leading to an infiltration of primed immune cells into the tumor and destruction of residual primary or metastatic tumor cells. This form of cell death which leads to the induction of the host immune system is referred to as immunogenic cell death (ICD) [162]. Unlike high-dose chemotherapy or radiotherapy with known immunosuppressive activity [163–165], PDT has shown the capacity to induce ICD through the recruitment of immune cells *via* DAMP release [166–168], resulting in immunogenic apoptosis [158]. This was initially noticeable in studies done in immunocompetent tumor models where long-term tumor cure was observed post-PDT [169]. More details on PDT-mediated ICD are mentioned in the section below.

## PHOTODYNAMIC PRIMING OF THE TUMOR MICROENVIRONMENT

Responses to PDT may be modulated to a large extent by varying the light dose, PS concentration, and DLI. While the tumoricidal ability of high PDT doses are well-established, recent evidence suggests that PDT effects are also detected remotely from the actual site of irradiation and with doses lower than those required to be cytotoxic. This effect is referred to as photodynamic priming (PD*P*) and may also confer increased immunogenicity [170] or enhance radio-, chemotherapeutic and RTKi susceptibility [41–43, 101] by priming multiple compartments in the TME. Although, the exact mechanistic differences observed in cells/tissues treated with PD*P*- and PDT-regimens are not well established; differences in tumor permeability and their transcriptomic, metabolomic, and proteomic profiles have been suggested [42, 171, 172]. Nevertheless, the impact of PD*P*-mediated modulation and sensitization to other therapies has also been reported [42, 173]. In this section, we briefly talk about the impact of PD*P* in modulating different components of TME and its effect on subsequent immune responses and sensitization to chemotherapeutic interventions.

### Photodynamic priming of innate and adaptive immune systems

Preclinical and clinical studies have demonstrated that PDT is capable of affecting both the innate and adaptive arms of the immune system. These immune stimulatory effects occur through its ability to induce ICD which increases the immunogenicity by priming immune cells in the TME *via* the release of DAMPs and TSAs [20, 65] (Fig. 5). In general, PDT generates DAMPs such as calreticulin, heat shock proteins (Hsp90, Hsp70, HspP60), high mobility group box-1 (HMGB1) and extracellular ATP [19, 65, 91]. DAMPs and cytokines (such as tumor necrosis factor (TNF)-α, interleukin (IL)-6 and IL-1β) released from PDT treated cells may cause acute inflammation and enhance infiltration of innate and adaptive immune cells at the irradiated tumor site [38, 39, 65, 158, 161, 174, 175].

PDT may enhance antigen presentation by professional antigen-presenting cells (APC), such as DCs, whereby TSAs are processed and presented to cells of the adaptive immune system; especially T cells. [20, 37, 174]. This was evident by a study from Gollnick *et al.* where Pf-PDT-generated murine breast tumor cell lysates showed the strongest stimulation of antitumor immunity *in vivo*, as compared to other radiation therapies [174]. When there is no inflammation, DCs remain in a quiescent state. During PDT-mediated release of DAMPs and subsequent inflammation, APCs mature and migrate to the draining lymph nodes (Fig. 5). This transition of DCs involves their activation *via* the upregulation of major histocompatibility class I and II molecules (MHC-I and MHC-II) and the costimulatory molecules CD80 and CD86 on their cell surfaces. Once DCs are activated they are efficient in priming CD4^+^ T helper cells and CD8^+^ cytotoxic T lymphocytes (CTLs) by the presentation of TSAs, and therefore initiate an effective adaptive immune response. Thereafter, antigen-experienced CTLs may migrate to the tumor site to attack the remaining and/or metastasized tumor cells [20]. The augmentation of anti-tumor immunity, by PDT, is dose-dependent. A sublethal low-dose of PDT (PD*P*) has been shown to enhance the infiltration of neutrophils and activated CTLs in the TME [170]. In addition, when the sublethal PD*P* is combined with a high dose, tumor-controlling PDT, a further enhancement of anti-tumor immunity and tumor control was observed [170]. This observation suggests the possibility of a PD*P*-based sensitization of the TME for subsequent enhancement of anti-tumor immune responses.

#### Generation of long-lasting immunological memory and enrichment of anti-tumor T cell repertoire.

Besides stimulating tumor-specific CTLs that are capable of destroying distant untreated tumors, PD*P* may also lead to the development of immunological memory against tumors, that can potentially prevent cancer recurrence [20, 65], however further investigations are needed to verify the validity of these conclusions. Moreover, it is hypothesized that PD*P* may lead to the enrichment of a pool of tumor-specific T cells [38, 65] (Fig. 5). T cell receptor (TCR) diversity is known to be a prerequisite for immune recognition of varied TSAs present in the TME [176–178]. This diverse pool of T cell clones would have a higher ability to find cognate TSA-bearing tumor cells [65]. The interesting connection to this notion may be suggested by the recent combinatorial applications of PDT with immunotherapy (discussed in a later section) against different tumor types showing long lasting treatment benefits in preclinical studies [179, 180]. Therefore, we postulate that PDT will be a good option to derive benefits from immunotherapy treatments especially in the case of immunologically ignorant (cold) or excluded tumor types [181] by making them more immunogenic and responsive to current treatment modalities.

### Photodynamic priming of the tumor microenvironment for subsequent therapy

Tumor cells generally grow in an uncontrolled fashion which often leads to the development of a microenvironment limited in nutrient and oxygen supply along with a build up of excreted metabolic waste. While cancer cells adapt to these harsh conditions through regulating signaling pathways, other cellular and acellular components of the TME also help in this adaptation process thus assisting the tumor cells to survive, proliferate, and metastasize. Together these components comprise a major physiological and mechanical barrier to conventional therapies, thus forming the basis of several recent studies on targeting components other than the tumor cells for enhancing cancer therapeutics [182–184].

**Fig. 6.**
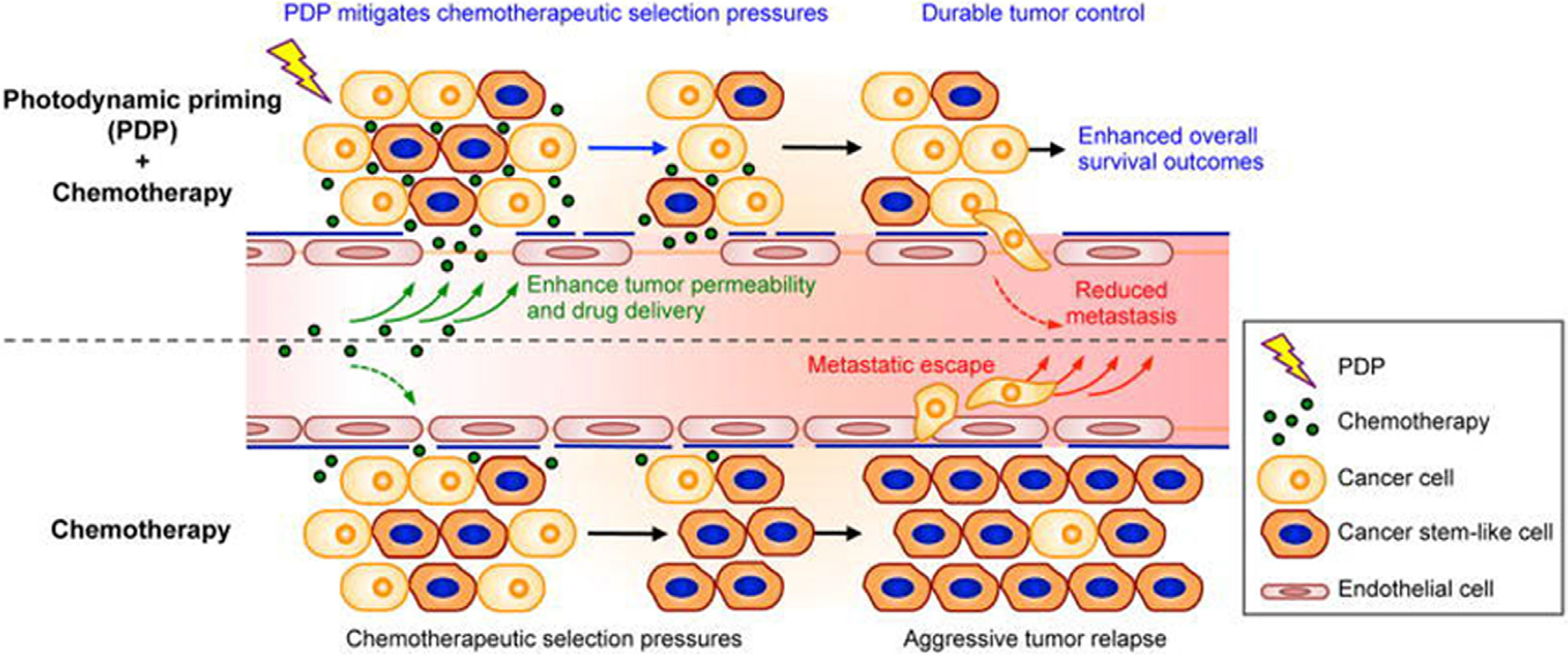
The impact of PD*P* on modulating multiple compartments in the tumor microenvironment. Firstly, PD*P* of tumor microvasculature and parenchyma simultaneously improves therapeutic agent accessibility and overcomes chemotherapeutic selection pressures. Secondly, sublethal PD*P* increases tumor permeability to enhance intratumoral accumulation of chemotherapeutic agents for a prolonged period of time. Thirdly, PD*P* attenuates the insidious surge of stemness marker expression that is typically observed after multiple cycles of chemotherapy. PDT-mediated immune enhancement discussed above is an example of such a priming process (PD*P*) where the impact reaches far beyond the cells directly targeted by PDT. Figure adapted from Huang *et al.* (2018) [42].

While the cellular mechanisms of PDT (PD*P*)-mediated resensitization of treatment resistant cells are well established and discussed elsewhere in this review, recent studies from our group have shown that PD*P* can prime multiple tumor compartments to enable a more potent and sustained anti-tumor chemotherapeutic effect [42] or dose reduction for improved tolerability [43]. Huang *et al.* demonstrated that PD*P* can mitigate drug delivery barriers in the TME to safely widen the therapeutic window of FDA-approved nanoliposomal irinotecan (nal-IRI) in a preclinical model of pancreatic cancer [42]. In this study, the combination of PD*P* with nal-IRI prevented tumor relapse, reduced metastasis and prolonged survival in mice models of orthotopic pancreatic cancer. PD*P* led to an increase of intratumoral drug accumulation, retention of drug in the TME and attenuation of surges in stemness marker (CD44 and CXCR4) expression, which mediate chemo-resistance observed often after multi-cycle chemotherapy (Fig. 6). A follow-up study by our group showed that when the same PD*P* was combined with vitamin D receptor activation, it led to the modulation of pancreatic TME to enable a 75% dose reduction in irinotecan while simultaneously preserving durable anticancer efficacy and improving tolerability [173]. These studies thus highlight the potential of PD*P* in TME modulation and may provide an attractive strategy to enhance the efficacy of conventional chemotherapeutic regimens.

## OPTICAL IMAGING FOR DIAGNOSTICS AND THERAPY GUIDANCE IN PDT

### Photosensitizer fluorescence for image-guided surgery

**Fig. 7.**
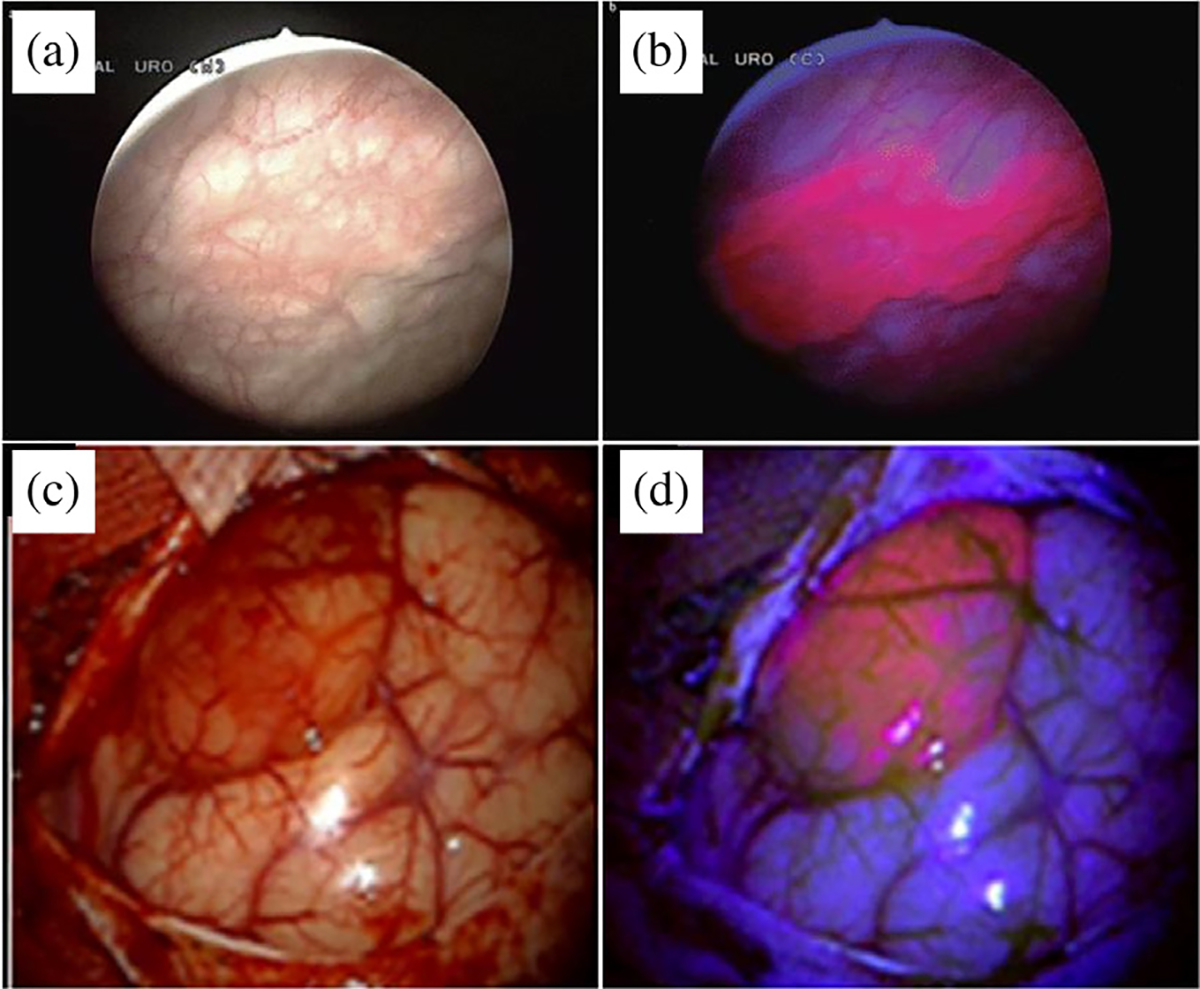
Bladder (a and b) and brain (c and d) tumor detection with regular white light (a and c), and fluorescence (b and d) imaging of PpIX. Images in (a) and (b) were acquired by an equipment for photodynamic diagnosis equipped with a light source (short-arc xenon lamp with a specially designed dielectric short-pass filter (375–440 nm)) for excitation light that can be transmitted through modified cytoscopes and lenses to maximally enhance the contrast between benign tissue and fluorescence from malignancies. Images in (c) and (d) were acquired by neurosurgical microscope equipped with a fluorescent 400 nm UV light module. Figures adapted from Zaak *et al.* (2005) [191] and Goraynov *et al.* (2019) [190].

As described earlier, most PSs display finite fluorescence quantum yields and thus can be used for imaging, therapy monitoring, and optimizing PDT dosimetry [13]. PSs have been utilized for their fluorescence for decades since the pioneering works of R. L. Lipson and S. Schwartz in the 1960s [25, 26, 185, 186]. The most impactful use of Photosensitizer Fluorescence Detection (PFD) to date has been for image-guided resection of brain and bladder cancers [187–191], which are now approved by regulatory authorities worldwide. These approvals were based on clinical trials using ALA-PpIX (5-aminolevulinic acid induced protoporphyrin IX conversion) based PFD for guiding resection, which demonstrated a significant increase in tumor-free survival as compared to white-light based tumor resection surgery (Figs 7a–7d) [187, 192, 193].

Apart from ALA, which is a precursor to the endogenous PS, PpIX, the administration of free PSs for fluorescence-based detection has been challenging due to their non-specific accumulation, thus, compromising signal to noise ratio [194]. In this context, several formulations with fluorophores/PSs conjugated to tumor-targeted molecules have been developed to improve specificity [195, 196]. Advances in the generation and biological use of therapeutic mAbs and technologies pertaining to the synthesis of antibody-drug conjugates have led to the development of several targeted PS conjugates (referred as photo-immunoconjugates; PICs) for image-guided surgeries and targeted PDT (more specifically referred to as photo-immunotherapy; PIT) [65, 197–200]. While PFD associated with PICs can be exploited to identify tumor margins during surgical resection and possibly detection of occult metastases, subsequent PIT of the resected tumor bed may assist in the treatment of residual microscopic tumor [201, 202], as discussed below. Currently, there are ongoing studies using targeted PFD for real-time molecular imaging in head and neck cancer resection surgeries [203].

Apart from PICs, where the PS is in an unquenched state and remains “ALWAYS ON”, homoquenched and heteroquenched target activatable constructs have also been reported [198, 204, 205], which rely on the conjugation of PSs in appropriate ratios to achieve self-quenching or the conjugation of a fluorescence resonance energy transfer (FRET) pair (in an optimal ratio) on the targeted molecules; which results in quenching of the fluorophore/PS [206]. Other procedures may involve the use of singlet oxygen/RMS quenching agents in combination with PSs to minimize non-specific toxicity [207]. Following target binding and dissociation of the quenched PS/fluorophore, fluorescence can be restored leading to an enhanced signal to noise ratio thus assisting in image-guided therapies.

### Detection and therapy monitoring in PDT

Efforts to develop real-time techniques to monitor response during and after treatment are being made with the aim of monitoring changes in tissues to inform therapeutic outcomes. Biological indicators of treatment efficacy include blood flow dynamics, blood perfusion, glucose metabolism, and oxygen monitoring. Apart from these biological indicators, measuring photobleaching and fluorescence dynamics of the PS are established methods of imaging PDT responses during treatment, the utility of which can be aided by online feedback capability, allowing real-time analytics to the clinician, further enabling immediate adaptations to treatment. While there are many ways of monitoring real-time treatment efficacy, treatment results, and potential for tumor regrowth, this section addresses a select few and will be organized below by separating monitoring techniques with a focus on optical imaging and spectroscopic-based methods, with additional separation by the physiological characteristic being monitored.

#### PFD-based optical imaging and detection.

**Fig. 8:**
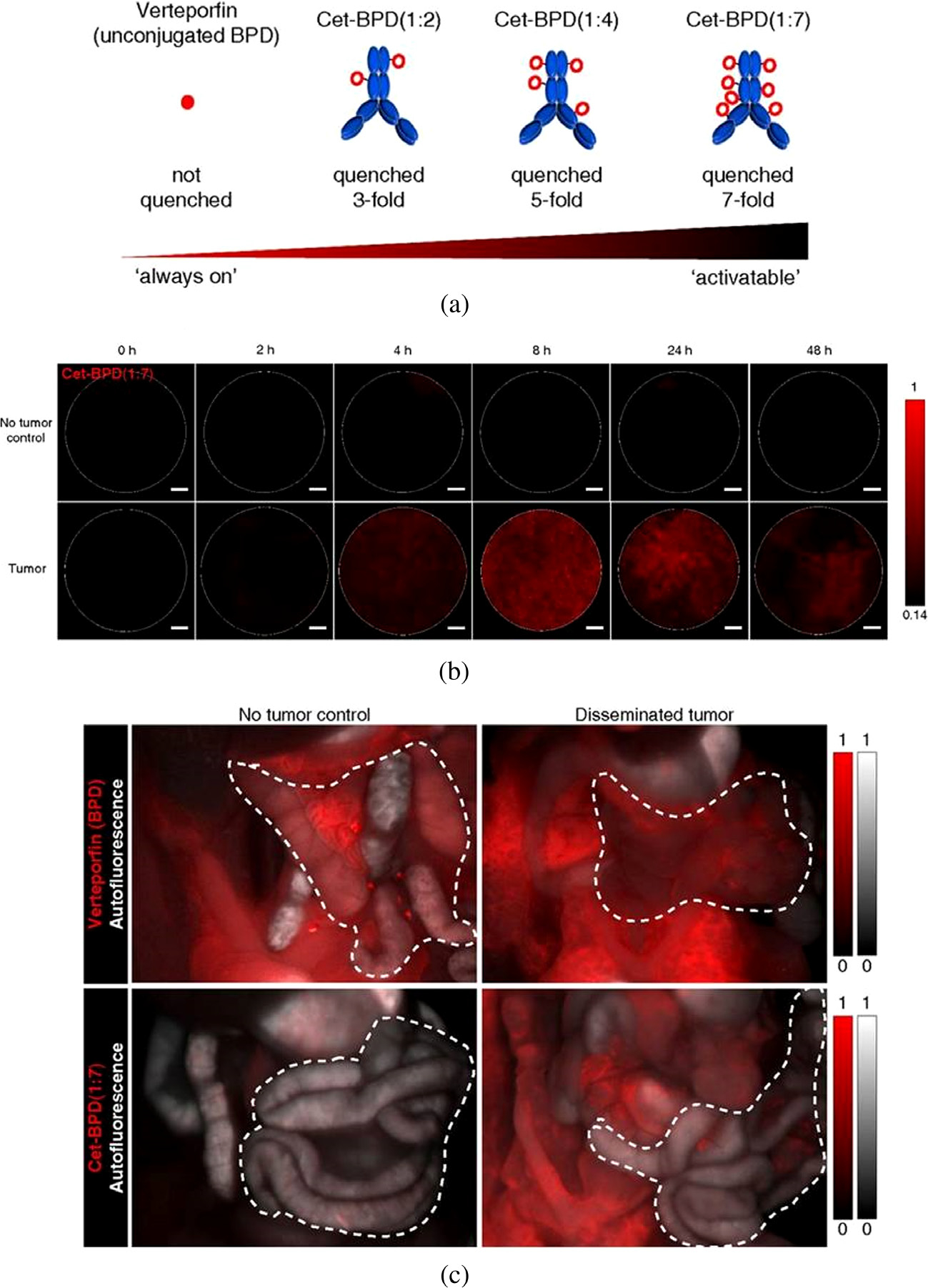
Target activatable fluorescence detection and photo-immunotherapy (taPIT). (a) PICs (Cetuximab-BPD) with different PS loading ratios, leading to varying BPD self-quenching efficiencies. (b) Longitudinal microendoscopic images of the peritoneal cavity demonstrate the efficacy of PICs to identify micrometastatic sites of ovarian cancer at 8–24 h post administration. (c) BPD fluorescence (red) and autofluorescence (gray scale) of the peritoneal cavity 2 h after free BPD or 48 h after PIC (Cetuximab-BPD) administration. Figure adapted from Spring *et al.* (2014) [198].

As described earlier, PFD associated with PSs has been extensively used for imaging applications and more recent advances utilize target activatable PFD methods. While targeted PSs usually achieve a high degree of selectivity and specificity for tumors, generation of PSs with target activatable fluorescence has shown further enhancement in the signal to noise ratio for diagnostic applications [197, 204]. A study in this context is the tumor-targeted activatable Photo-Immunotherapy (taPIT) using the FDA approved anti-epidermal growth factor receptor (EGFR) antibody (Cetuximab; Cet) conjugated with the PS; BPD in self-quenching ratios [198, 208], evaluated in preclinical models of ovarian cancer micro-metastasis. As demonstrated in this study, through a Cet to BPD ratio of 1:7 (homoquenching ratios), it was possible to impart tumor activatable properties to the PICs (Fig. 8a), with subsequent longitudinal hyperspectral fluorescence imaging of the bowel demonstrating activation of the probe 8–24 h post-administration (Fig. 8b) which was significantly higher, as compared to the non-targeted PS (Fig. 8c). This study puts forward a case for PFD-based diagnostics and treatment of ovarian cancers, where residual microscopic tumors post-treatment has been a major cause of recurrence and mortality [209].

Apart from imaging tumor tissues, targeted antibody conjugates can also be valuable for PFD-based imaging of molecular markers. In this context, Chang *et al.* demonstrated *in vivo* optical molecular imaging utilizing an anti-VEGF antibody conjugated with fluorescent dye to image vascular endothelial growth factor (VEGF) expression in tumors following PDT and were able to show, quantitatively, the changes in tumoral VEGF concentration and release of VEGF into the extracellular matrix following PDT [210, 211]. Furthering the clinical utility of molecular imaging combined with targeted immunoconjugates, Zhong *et al*., [212] used tumor-selective fluorescence contrast to provide accurate and timely information of the treatment response during, and immediately following, treatment. Fiber-optic fluorescence imaging of BPD before and after PDT, in preclinical studies, demonstrated a method for obtaining immediate response feedback of tumor destruction and prediction of tumor regrowth following treatment. Online monitoring of PDT response has been further characterized by Garcia *et al.* [213] with NIR fluorescence signal of PpIX used to monitor PDT (which notably also provides simultaneous therapeutic effects, as with BPD) of skin cancer. Despite several encouraging preclinical studies, PFD is not a popular option in clinical settings for diagnostic applications mainly due to the existence of fluorophores which have much higher fluorescence quantum yields and hence sensitivity. However, the advantage provided by PSs in terms of their theranostic potential is unmatched where diagnosis and therapy can be achieved using a single agent.

In addition to the studies using homoquenched systems discussed above, an interesting approach involves heteroquenched conjugates which, in principle could provide higher specificity. Although, heteroquenched antibody-based targeted systems have not been demonstrated for PSs yet, a study by Obaid *et al.* utilizing heteroquenched fluorophores, and achieving a ~9.8 fold increase in fluorescence signal, post-activation [204], can be adapted for PS-based fluorescence detection as well. More recent formulations of PSs, in homoquenched or heteroquenched state, involving the use of nanoconstructs have been reported [214–216]. These systems have shown promise in imaging tumors in several preclinical studies. Nanoconstructs, owing to their ideal size, accumulate in tumor tissues (due to EPR effect) after systemic application and can be imparted with TME activatable features (responsiveness to pH, hypoxia, and redox potential) to trigger PS activation for imaging and PDT [215–217]. Although, still in its infancy, such constructs hold promise as diagnostic and therapeutic (theranostic) agents and are discussed in more detail later in this review.

#### Spectroscopic measurements of photobleaching for PDT dosimetry and monitoring efficacy.

The dual-function nature of PDT, in that the photoactivatable drug, PS, both releases RMS to act on a tumor (which can also be spatially controlled by light) and can also be used as a fluorescence reporter, enables the use of PSs to act as theranostic agents. PS uptake prior to PDT and PS fluorescence reporting during and after PDT can be used as a tool to both monitor PS dosage, as well as signify treatment efficacy and potentially predict regrowth. Photobleaching has been used to correlate dose and edema induced in the tissue and can be used both to predict treatment efficacy, as well as guide dosimetry in PDT [218, 219]. It has been pursued for dosimetry for a long time and early studies by Van den Bergh and team formed the basis for many subsequent studies [220–222]. Active monitoring of PpIX photobleaching, *i.e.* for dosimetry purposes, has been used in clinic, as demonstrated by Pogue *et al*., in which a probe light is applied to the tissue during PDT and is intermittently turned on for fluorescence measurements. These studies were able to show that active monitoring can be used to measure PS uptake and convert the probed fluorescence intensity to PS concentration, thus potentially providing important metrics for clinicians during PDT [223–225]. Further, studies have used photobleaching to support the hypothesis that light fractionation in ALA-PpIX-based PDT during esophageal treatment increases efficacy [226]. Additional studies have used PpIX photobleaching in clinical settings in *e.g.* basal cell carcinoma [227], and most recently in monitoring ALA-PDT in oral cancers [228]. These observations are consistent with the earlier reports by Van Der Veen *et al.*, in which photobleaching of PpIX was utilized as a method to study the benefits of a second illumination in ALA-PDT [229]. Applying ALA-PDT to nude mice, the group was able to take advantage of the dual nature of ALA as a PS to both perform PDT and measure the fluorescence spectrum/intensity of PpIX to show that an additional illumination may increase PDT efficacy with topical application of ALA due to synthesis of PpIX following initial illumination [229]. In all, photobleaching is an elegant method to take advantage of the dual reporting and therapeutic nature of a PS in PDT treatment and has been shown successfully to provide real-time information in humans.

While photobleaching, in principle, could be a useful treatment monitoring tool, there are aspects that can be further improved. Robinson *et al.* demonstrated that the correlation of photobleaching to PDT-induced damage involves a complex relationship between fluence rate, PS concentration, and local oxygen concentration [230]. Fluorescence may only address the broad emission from all states of a PS, whereas more controlled experiments, such as monitoring of singlet oxygen, could report on a single component of the PS *viz.* the active state that is largely responsible for damage incurred in PDT. This might address the complicated nature of oxygen in terms of dosimetry *via* PS fluorescence. Efforts to monitor fluorescence, while removing environmental effects to the PS, have been made using fluorescence lifetime measurements due to the inherent sensitivity of lifetime (lifetime is an intrinsic property of the PS and independent of concentration, sample absorption, sample thickness, measurement time, and photobleaching). Fluorescence lifetime imaging of mice undergoing HpD-PDT and time-gated fluorescence spectroscopy combined with post-processing revealed spatial distribution of HpD fluorescence in the tumor [231]. This *in vivo* study was performed by measuring the spatial distribution of HpD fluorescence lifetime using nanosecond pulsed laser excitation combined with time-gated camera detection and mathematical processing. This, however, has not been tested in humans yet. Barriers to implementing fluorescence lifetime measurements in clinic are largely based on limited speed and ease of use in a clinical setting, where expensive and/or custom-made systems are not ideal. With the increase in cheap and compact instrumentation, time-resolved fluorescence measurements may hold promise for future clinical applications. Laubach *et al.* published the first report of monitoring singlet oxygen luminescence in human subjects during PDT, demonstrating the correlation of singlet oxygen levels during PDT with post-PDT photobiological response [232]. Mallidi *et al.* built on this work [233] to report the use of discrete single oxygen-based dosimetry measurements compared against discrete PS fluorescence-based dosimetry to predict erythema resulting from ALA-PDT in humans. The authors hypothesized that PS-fluorescence based measurements allow distinctly different predictive capabilities than singlet-oxygen based metrics and determined that PS-fluorescence monitoring had better performance for dosimetry. Further, the authors suggest that, while singlet-oxygen based dosimetry provided higher sensitivity and correlated well with phototoxic response of the skin, PS-fluorescence based metrics provided better overall prediction of treatment response in the subjects. Optical systems have been adapted for near-infrared detection of ^1^O_2_, tested *in vitro,* as done by Kim *et al.* [234], as well as *in vivo* in animal tumor models [235–239], using fluorescent probes during PDT for monitoring. Despite the suggested correlation of singlet oxygen luminescence with treatment outcomes, the necessity of high-sensitivity detection systems (due to low luminescence signal from ^1^O_2_), and complications of ^1^O_2_ stability *in vivo*, equipment adaptations for online monitoring and analysis remains elusive, with further work needed to translate these techniques to humans. Furthermore, the significance of ^1^O_2_ measurement remains complex given PDT effects can be induced by non-oxygen dependent pathways. In addition to the direct measurement of luminescence, a recent study, at the time of writing this review, an approach to monitoring singlet oxygen, proposed by the Pogue group was explored *in vivo* in tumor-bearing mice [240]. The technique takes advantage of the singlet oxygen feedback delayed fluorescence (SOFDF) mechanism, intrinsic to many common PSs. Overcoming the need for additional molecular probes for ^1^O_2_ measurement and enabling detection in the visible spectral region, among other benefits, present an interesting novel method, and although not yet shown in a clinical setting, will likely continue to develop as a valuable and exciting part of parallel monitoring techniques.

#### Blood oxygen saturation as a marker for PDT efficacy.

**Fig. 9.**
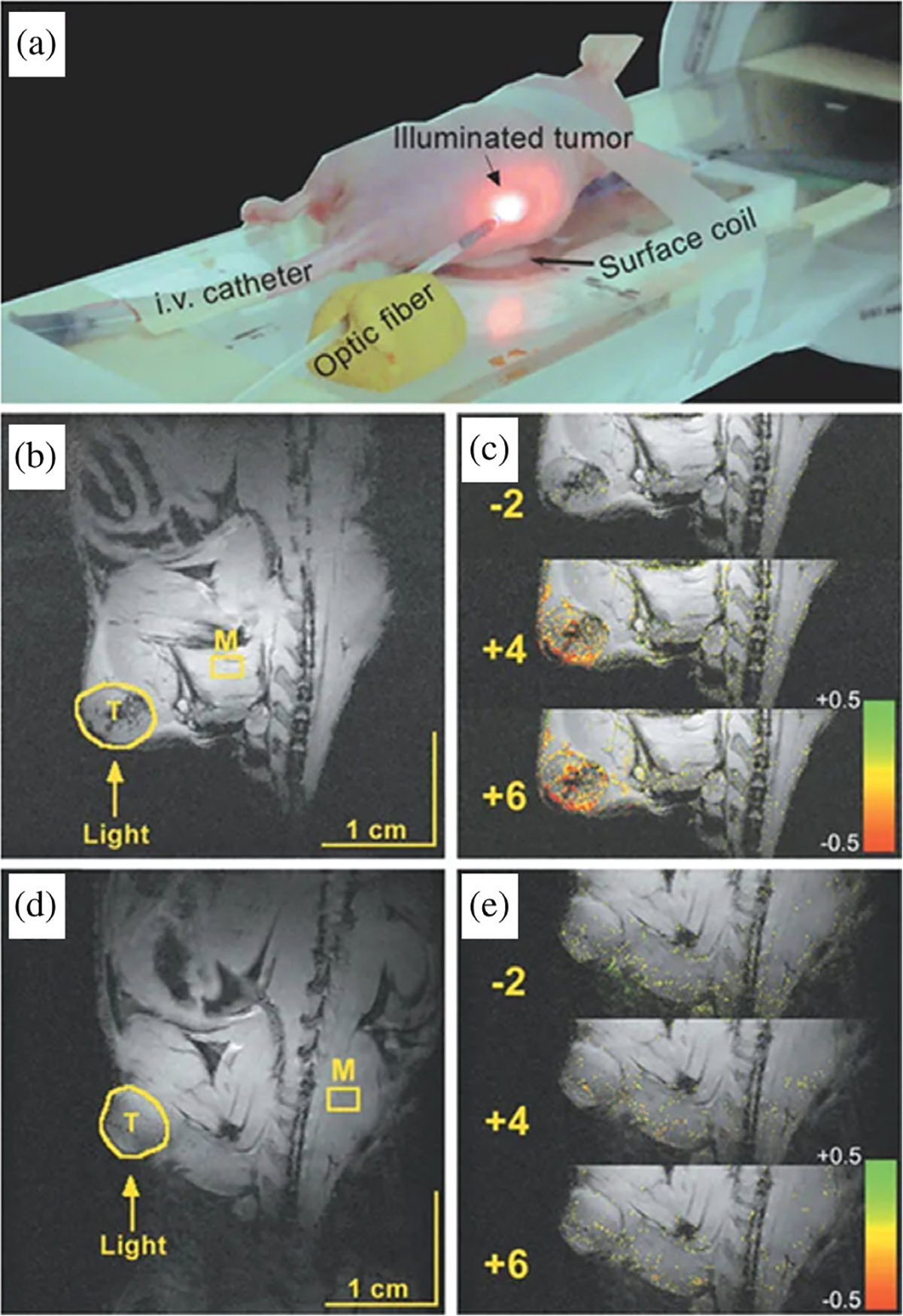
Experimental setup (a), anatomical images of pre- and post-PDT treated (b) and control (d) mice. BOLD-MRI intensity of control (c) and treated mice (e). Color coding represents ratio of signal intensity over baseline. Figure adapted from Gross *et al.* (2003) [251].

As type II PDT involves light-induced consumption of molecular oxygen to produce reactive molecular species, changes in blood oxygen saturation (StO_2_) are a natural response to PDT and it can be hypothesized that changes in StO_2_ can be indicative of PDT efficacy and potential tumor regrowth. Measurements of StO_2_ can be done in multiple ways; with some of the earliest *in vitro* work done by Reed *et al.* [241], where oxygen partial pressure was measured before and after HpD-PDT. Also, *in vivo* work by Tromberg *et al.* [242], showed depletion of oxygen and disruption of tumor blood flow, as measured *via* transcutaneous oxygen electrodes, thus providing the first non-invasive real-time measurements of tissue oxygenation during irradiation. Although oxygen consumption can conceptually provide valuable information on PDT progress, technical aspects of oxygen measurements *via* electrodes present inherent drawbacks such as low spatial accuracy, low signal-to-noise, and environmental effects on the probe [243]. Given the differing optical properties of oxy-hemoglobin and deoxy-hemoglobin, later methods developed upon quantification of tissue absorption and tissue scattering properties. Total StO_2_ was measured using optical data by Pham *et al.* [244], in which real-time non-invasive quantification of physiological effects during PDT were monitored with near-infrared spectroscopy and correlated to therapeutic efficacy. There are continued efforts towards online monitoring of PDT efficacy and tumor recurrence predictions with StO_2_ measurements and resultant blood flow response *via* diffuse correlation spectroscopy [245–249]. Blood oxygenation level-dependent contrast magnetic resonance imaging (BOLD-MRI) was first shown for measurements of deoxygenated human blood *in vivo* by Ogawa *et al.* [250], and has further developed into a viable method for imaging oxygenation and blood flow which, given the importance of vasculature function in tumors, as well as oxygen consumption related to PDT treatment is an excellent resource for assessing PDT progress. Gross *et al.* [251] demonstrated BOLD-MRI for real-time monitoring of TOOKAD^®^-PDT efficacy in a solid M2R mouse melanoma model and provided information on vascular shutdown *via* a decline in perfusion rate and changes in MRI signal (Fig. 9). BOLD-MRI has since been further developed for online measurements *in vivo* [252, 253].

As vascular perfusion and blood flow have been shown to increase immediately after PDT and return to base values shortly after treatment, PDT-induced vascular changes and the rate of blood flow can be measured to monitor treatment or to determine ideal drug-light intervals. Chen *et al.* used a combination of fluorescence microscopy (for PS localization imaging) and laser Doppler to monitor arrest in blood flow following PDT treatment [254]. Using a radiation-induced fibrosarcoma (RIF-1) tumor model in C3H/HeJ mice, a verteporfin (1 mg/kg)-based PDT (DLI: 15 min to 3 h; irradiance of 120 mW/cm^2^ and total light dose of 100 J/cm^2^), achieved through a diode laser system (λ = 690 nm), led to a finding that a shorter drug-light intervals with verteporfin-PDT led to more significant damage to the tumor vascular bed and overall tumor control [254]. In addition, laser speckle imaging [255–257], and optical coherence tomography and angiography [258] provide non-invasive methods for blood flow measurements during PDT.

**Fig. 10.**
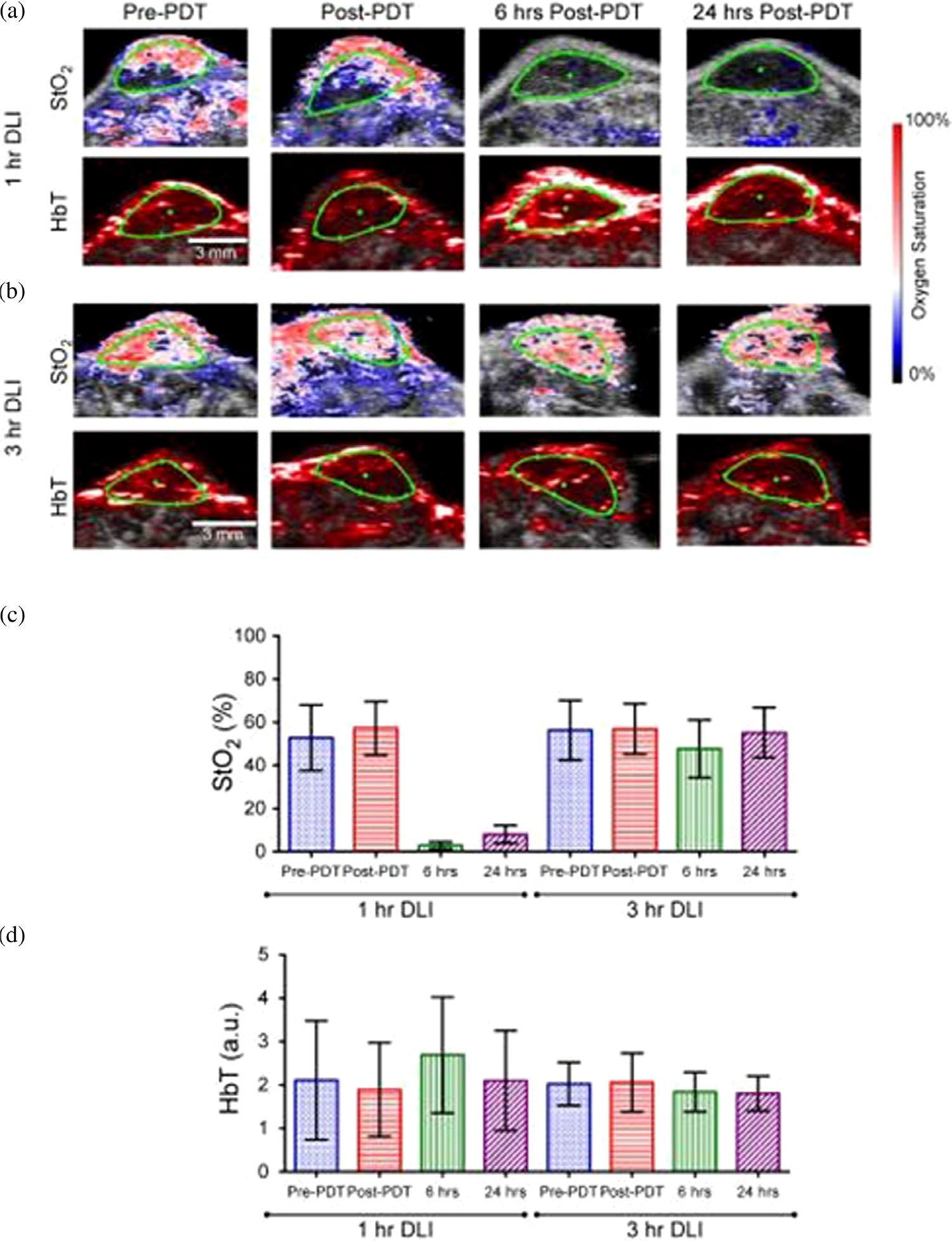
(a), (b) Ultrasound and photoacoustic images of oxygen saturation (StO_2_) and total hemoglobin (HbT) demonstrating mapping of hypoxic (blue) and oxygenated (red) regions in a murine model of glioblastoma. 1 h DLI and 3 h DLI refer to initiation of irradiation 1- or 3-h following PS-application. Green region outlines tumor region identified using ultrasound (c), (d) Mean StO_2_ and HbT values at pre-PDT, post-PDT, 6 h, and 2 h. Figure adapted from Mallidi *et al.* (2015) [16].

Efforts to improve upon clinically realistic online monitoring of PDT efficacy include adaptations in technology already in-use in clinic, with low-risk changes to optical instrumentation such that mapping of StO_2_ and correlation with standard therapeutic prediction becomes translatable from lab to clinic. Mallidi *et al.* demonstrated a clinically relevant photoacoustic imaging (PAI) based method that could predict tumor efficacy by measuring oxygenated and deoxygenated hemoglobin post-PDT (Fig. 10) [16]. The authors demonstrated that ~95% and ~85% increase in tumor StO_2_, 6 h and 24 h post-PDT, respectively, were predictive of tumors responding positively to treatment, where no changes in StO_2_ were observed for non-responding tumors. Using this system, a “prediction map” within 24 h post therapy could assist in deciding on secondary intervention. PAI-based systems present an option for “mapping” of a tumor, and showcase options for monitoring PS tumor uptake as well as providing personalized feedback of dosing and concentration [259, 260], or vasculature changes [261, 262].

#### Metabolic changes as a marker for monitoring cell death following PDT.

Cancer metabolism and changes to redox state in cancer cells is a useful indicator of disease progression and response to therapy. Measuring dynamic changes in metabolism, following PDT treatment, may be expected to provide valuable spatiotemporal metrics of cancer tissues. Of the initial studies on continual measurements of metabolic activity in PDT treatment, the Lecomte group demonstrated the use of PET imaging to study tumor response and mechanism of action of PSs. The authors employed two different PSs with different mechanisms of action; Pf and disulfonated aluminum phthalocyanine (AlPcS). The tumor perfusion agent — 2-deoxy-2-[(18)F]fluoro-D-glucose (FDG) with PET was used to monitor tumor metabolism following PDT treatment. The authors found significant reduction in FDG signal in Pf-PDT treated tumors early after PDT, whereas AlPsC-PDT treated tumors demonstrated a slower, more gradual decrease in FDG, reflecting slower decrease in tumor activity, with fewer vascular effects and rather more direct tumor cell inactivation. Alternatively, the initial decrease in FDG seen in Pf-PDT treated tumors indicates stronger indirect vascular effects initially following treatment [263–266].

Mitochondrial function as an indicator of cell metabolism following treatment is an interesting parameter to monitor; in that nicotinamide adenine dinucleotide (NADH) has a distinct fluorescence signal and excited state lifetime, which can be probed for following PDT response, as shown by Pogue *et al.* in 2001 [266]. A 22% reduction in NADH fluorescence signal after verteporfin-based PDT was seen, compared to no significant change in fluorescence signal in non-treated controls, thus providing a dosimetric measurement of PDT-induced cell death. Recent work by Broekgaarden *et al.* [267] monitored mitochondrial autofluorescence intensity with high-throughput fluorescence imaging and resultant redox states of cells and tissues were determined *via* ratiometric analysis of oxidized and reduced states of NAD(P)H/NAD^+^ and FADH_2_/FAD. Although more work has to be done on adapting this method in clinical online measurements for deriving direct feedback regarding PDT efficacy, this method presents a novel technique, adaptable to heterogeneous cancerous tissues [268].

## NANOTECHNOLOGY AND TARGETING IN PDT

**Fig. 11.**
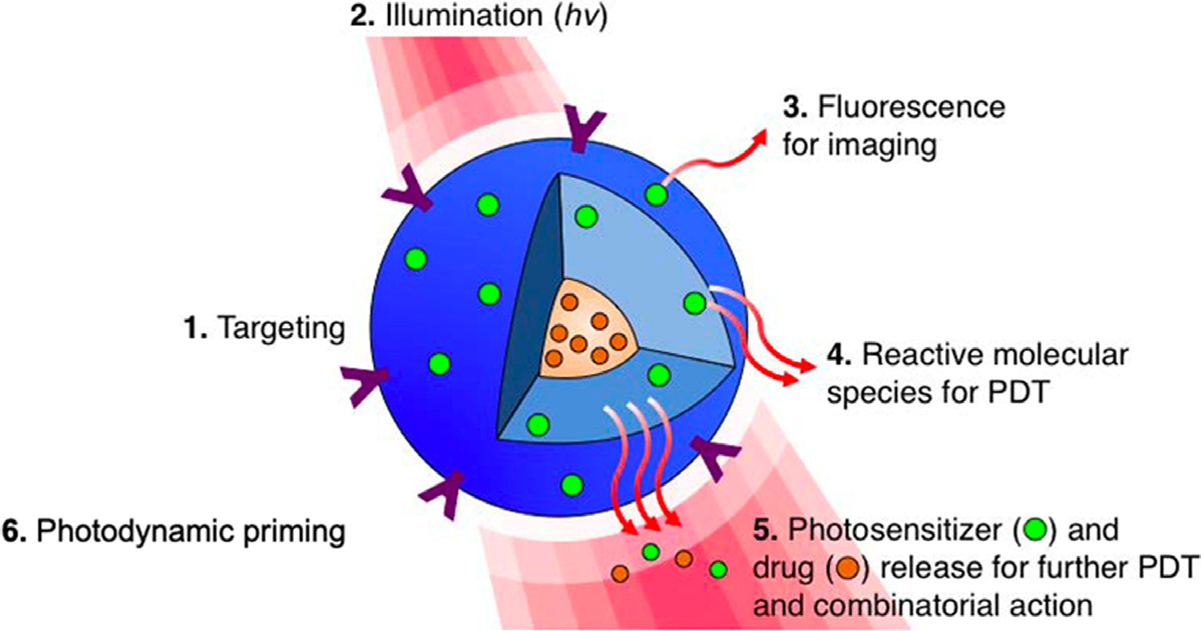
Schematic diagram of a targeted multi-agent nanoconstruct. (1) The surface is grafted with targeting moieties to endow molecular selectivity. (2) Light activation/illumination of the photosensitizer can be used for (3) fluorescence-based imaging (4), photochemical generation of cytotoxic reactive molecular species (RMS) for PDT (5), sequential, controlled release of synergistic therapeutic agents for combinatorial cancer therapy, and for (6) photodynamic priming of tumor microenvironment. Figure adapted from Huang and Hasan (2014) [24].

Since the excitement following the approval of Visudyne for the treatment of age-related macular degeneration (AMD), the clinical use of PDT appears to not have increased at the pace expected despite several regulatory approvals and ongoing clinical studies [269–271]. While the reasons for the limited clinical use of PDT are complicated, advances in what we call “off shoots of PDT” such as nanotechnology and photochemical internalization (PCI) open up new horizons and offer hope. Nanotechnology has the potential to provide more robust paradigms for diagnostic and therapeutic interventions of diseases. Combined with photodynamic activation, nanotechnology assists in developing advanced PS/drug formulations, targeting therapeutics, controlled delivery, and drug release for the treatment of various diseases (Fig. 11) [272–275]. These are unique attributes of PDT using nanotechnology and perhaps other externally activated therapies. The same construct provides not only a PS delivery mechanism but also light-triggered release of one or more agents at the “same-place same-time” thus impacting the tumor maximally for combinatorial effects. Moreover, formulating PSs through nanoconstructs also addresses several challenges related to the physiochemical properties of PSs, their systemic distribution, low selectivity, and certain TME factors such as low oxygenation [276–278]. A wide variety of nanocarriers such as liposomes, polymeric micelles, polymeric nanoparticles, and inorganic nanoparticles have been investigated for the encapsulation of hydrophobic and hydrophilic PSs in an attempt to enhance their delivery to the disease site [279, 280]. These formulations rely on the passive accumulation in the tumor through the EPR effect [281] and degrade at desired sites thus allowing for PS release. Similar to some drugs, the use of nanoformulations for PS delivery has been promising in minimizing phototoxicity associated with PS localization in healthy tissues.

In the past 30 years, liposomes and lipid-based nanoformulations have been extensively explored for use in PS delivery [278, 282, 283], of which Visudyne (liposomal formulation of a hydrophobic PS–BPD-MA) was the first formulation for PDT, approved as first-line treatment. It continues to be used for patients with AMD along with the treatment of other non-cancerous and cancerous pathologies [284–286]. Another important feature of liposomal formulations is their ability to simultaneously encapsulate hydrophobic (including PSs), hydrophilic drugs and imaging agents in different compartments (Fig. 11). While the synthesis of these nanoformulations is not simple; excellent work by Drummond and colleagues has shown this to be possible [287, 288]. The ability to be captured in different compartments and be released upon photodynamic trigger allows the physicochemical properties of these drugs to be retained. As PDT synergizes with several other therapies and has been established to reverse chemoresistance in certain cancer types, nanotechnology-based platforms, comprising mechanism-based synergistic combinations, hold promise as carriers to achieve synchronized pharmacokinetics of multi-drug formulations and enhance therapeutic outcomes [41]. Moreover, nanoformulations can be engineered by conjugating multiple functional ligands, including antibodies, peptides, folic acid or glycoproteins (transferrin) to enhance tumor targeting, improve therapeutic outcomes and minimize off-target effects [276, 278, 280, 289]. This aspect becomes important as we gain more insights into the heterogeneity observed in tumors and realize the emerging role of non-tumor cells in the TME [290]. Designing formulations that can efficiently navigate in the TME and exclusively target tumor cells is an area of active research. Recent promising studies, related to targeted photomedicine, utilizing antibody conjugated constructs, sparing fibroblasts [40], and immune cells [291], in the TME, yet effectively eliminating tumor cells, have been reported. Encouraging findings on multi-faceted theranostic platforms, emphasizing the synergy between photochemistry and nanomedicine, will contribute to drive nanotechnology-based targeted therapeutics, for PDT in medicine.

### Targeted delivery of nanomedicines for photodynamic therapy

Nanomedicine-aided PDT exhibits enhanced specificity for tumor cells and minimal side-effects coupled with effective photo-destruction of cancer cells both *in vitro* and *in vivo.* While some PSs have inherent selectivity towards tumor cells [292], irradiation of target sites post PS administration provides further control to confine PDT responses. As mentioned previously, tumor selectivity can be improved by targeted agents that can carry therapeutic payloads from the site of administration to the tumors, leading to selective accumulation of therapeutics within tumor cells, thus improving the bioavailability of the PS. As non-specific PS accumulation and the associated phototoxicity still remains a major challenge in PDT, exploitation of tumor surface markers, including growth factor receptors (VEGFR, EGFR) [293–296], transferrin receptors (TfR) [297], folate receptors (FR) [298], low-density lipo protein (LDL) receptors [299], glucose transporters [300] and integrin receptors [301] for active targeting offers a viable option to minimize these side-effects. Moreover, light-activatable nanomedicines possess combined benefits of tumor activation and delivery of potent antitumor therapeutics with the potential of PDT.

#### Vascular-targeted photodynamic therapy.

Solid tumors are highly heterogeneous and dependent on angiogenesis for growth and metastasis. Antiangiogenic therapies [302, 303] have been developed for well-established vascular targets including VEGF and its receptors [304, 305], suggesting the use of tumor vasculature specific and tumor-homing peptides to inhibit tumor-induced angiogenesis [302, 303]. A major determinant of PS-based toxicity is the DLI. As the PS travels from the vasculature to the TME and is subsequently internalized in tumor cells, short DLIs lead to predominantly vascular effects while illuminations with longer DLIs favor tumor cell killing. In this context, the destruction of tumor neovasculature by vascular targeted PDT (vPDT) is compelling and can be attained to a large extent by managing DLIs or temporal control of light administration [306–309]. This forms the basis of the best-known application of PDT in the treatment of AMD which for several years was the only first-line therapeutic option for any application of PDT. Vascular-targeted PSs such as TOOKAD^®^ (WST09), a photosensitizer from the bacteriochlorophyll derivatives family has received clinical approval in several countries including Mexico, the European Union, and Israel for the treatment of early prostate cancer. This molecule requires an excipient; Cremophor^®^ [309, 310], for solubilization during clinical and preclinical use, with some concerns related to toxicity. A newer version of the same family of photosensitizers, called TOOKAD^®^ Soluble (WST11, padeliporfin; palladium bacteriopheophorbide monolysotaurine), requiring no excipients, is being investigated preclinically [311, 312] and clinically (NCT01310894, NCT03315754, NCT00707356, NCT00975429) [313–317] for prostate cancer [318]. The other photosensitizer formulation approved for vPDT is lipid anchored BPD (Visudyne), currently in clinical use. The proposed mechanisms for the two molecules are quite different. While BPD (Visudyne) is believed to act *via* the classical “type 2” mechanism, elegant studies by Scherz and colleagues [319, 320] showed that TOOKAD^®^’s primary mechanism of action is *via* the “type 1” mechanism leading to the generation of superoxide (O_2_^−•^) and hydroxyl (OH^•^) radicals [321, 322]. They demonstrated that post-irradiation, reperfusion of the irradiated region with oxygenated blood could occur due to the release of the vasodilator - nitric oxide (NO^•^). The interaction of the generated NO^•^ with the photo-generated O_2_^−•^ and OH^•^ may further enhance photodamage and lead to vascular occlusion [323]. Strategies to enhance and understand the anti-vascular effect of PDT, through the PS Benzoporphyrin derivative, have primarily focused on the temporal variations of the administration of light [324–326], which demonstrate enhanced vPDT efficacy. Apart from this, overexpressed receptors specifically located on angiogenic endothelial cells are also promising molecular targets for PDT. Direct targeting of the tumor vasculature not only leads to inhibition of tumor growth and metastasis by vascular destruction, it could possibly facilitate liposomal distribution by overcoming diffusion barriers in the tumors [327]. An important feature of vPDT, through nanoconstructs, is the ability to deliver angiogenic inhibitors and photophysically confine their action in the tumor vasculature thus minimizing the associated toxicity.

#### Enhancing combination therapy outcomes by the use of nanotechnology and PDT.

Heterogeneity of cancers combined with their ability to acquire resistance to standard therapies remains a major challenge in cancer therapeutics. The PD*P* attribute of the PDT process suggests that a combination of PDT and established anticancer therapies such as ionizing radiation and/or chemotherapy provides potentially improved treatment options. Chemotherapeutic efficiencies are often limited by low tumoral uptake of anticancer drugs [328], nonspecific drug distribution, and multidrug resistance. In this context, PDT in combination with several chemotherapeutic drugs (cisplatin, gemcitabine, irinotecan, carboplatin, cabozantinib/XL184) has been shown to reverse chemoresistance and increase treatment sensitivity even at lower drug doses in preclinical models and also in clinical settings [41, 49, 56, 100, 101, 173, 329, 330]. Perhaps, an important study in this context, worth mentioning, is the reversal of cisplatin resistance observed in *ex vivo* cultures of human ovarian cancer cells, by pretreatment with CA125 targeted PDT [331]. Such resensitization of treatment resistant tissues, is particularly attributed to the ability of PDT to directly target and inactivate anti-apoptotic proteins (Bcl-2 and Bcl-xL), often over-expressed in treatment resistant tumor cells. While, the synergistic potential of PDT and chemotherapeutic drugs has been long established, nanotechnology-based platforms have made possible the simultaneous loading of multiple therapeutic agents, to achieve a synchronized pharmacokinetic profile and enhance treatment outcomes. Apart from this, PD*P* with its ability to modulate tumor microenvironment has the potential to enhance drug uptake and distribution in the tumors. Huang *et al.* presented preclinical evidence of overcoming treatment barriers through PD*P*, enabling potent and sustained antitumor activity of nanoliposomal irinotecan (discussed in detail in a previous section) [42]. The photo-initiated approach significantly reduced metastases and prolonged survival in orthotopic models of human pancreatic cancer cell lines [42, 332]. The overall long-term success *in vivo* was attributed to a combined priming of the tumor microenvironment by PD*P* enabling high doses of chemotherapeutic agent delivery while cancer and stem cells were both killed agnostically. It should be noted that studies [42, 332, 333], which evaluated combination therapies on cancer stem cells/cancer cells and PDT have used established cell lines and not patient derived tissues, which makes the significance of these observations ambiguous. The heterogeneity observed in human tumors is lost when generated from single cell lines and therefore the impact on the loss of stem cell markers from tumor is not completely obvious. On the other hand, these cell lines are not monoclonal and so, there is always heterogeneity in the molecular profiles.

**Fig. 12.**
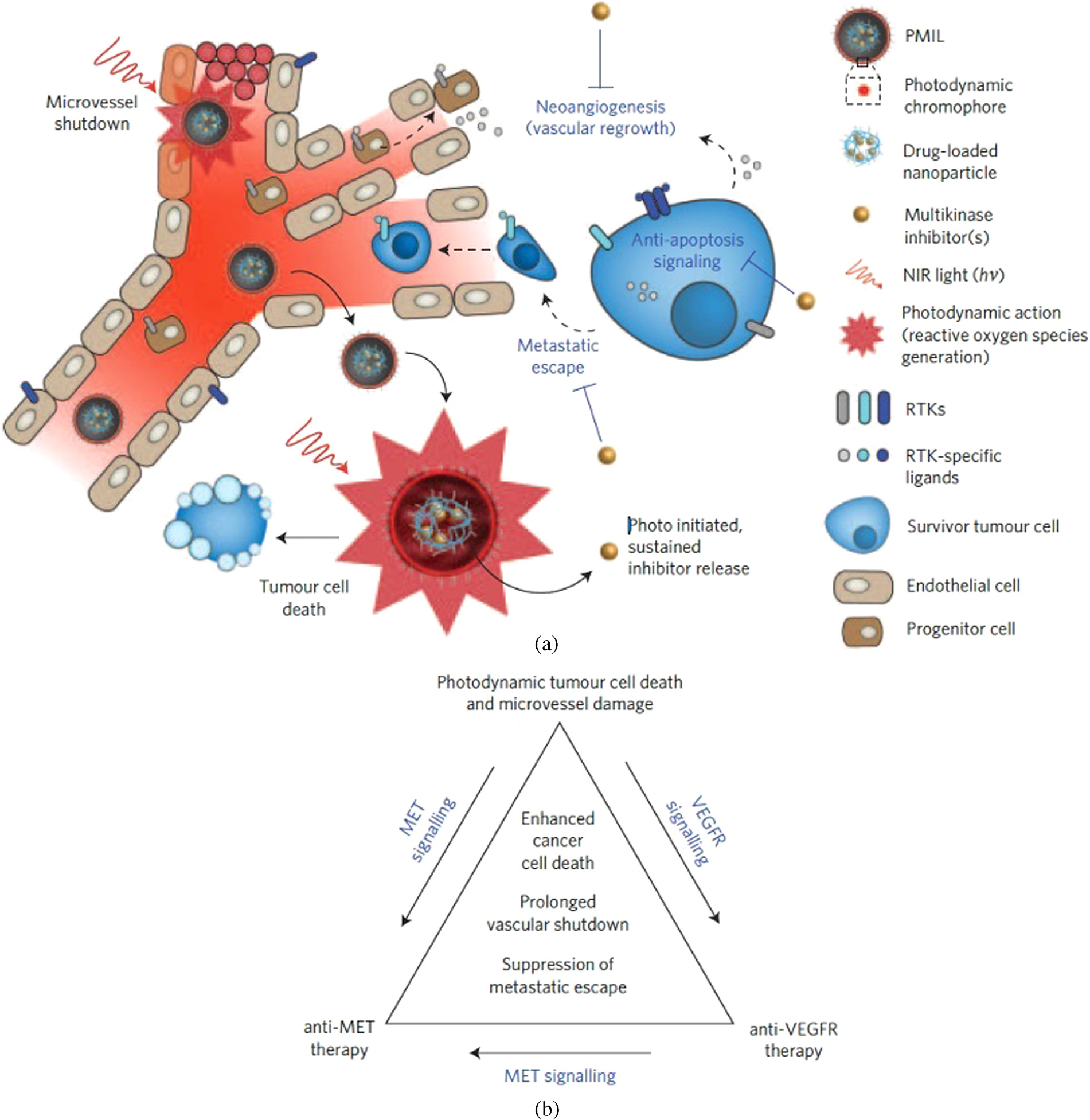
Concept of spatiotemporal-synchronized combination therapy using PMILs. (a) NIR light activation of PMILs within the tumour microvasculature for simultaneous neovascular damage, tumor cell apoptosis and necrosis and liposomal disruption to initiate sustained multikinase inhibition. (b) Schematic of a three-way interactive combination therapy with photodynamic tumour cell and microvasculature damage and inhibition of treatment escape pathways. Figure adapted from Spring *et al.* (2016) [41].

The significance of combination therapy is perhaps best realized when a single nanoconstruct contains a combination of therapeutic agents. This allows for spatio-temporal control “right-place right-time” over chemotherapeutic drug delivery made feasible *via* a photo-trigger leading to adequate intracellular release whilst reducing systemic drug exposure and associated toxicities [41]. In general, as shown in Fig. 12, RMS sensitive nanocarriers are ruptured to achieve tunable drug release profiles [41, 334]. Spatiotemporally controlled light-triggered release of encapsulated cargo [335, 336] could be induced by the inclusion of RMS sensitive lipids in liposomes, thioketal linkages in polymer nanoparticles, *etc*. Liposomal formulations of various size, compositions and surface characteristics (as described earlier) have been used either to incorporate a wide range of anticancer agents such as doxorubicin [337], irinotecan [287], and mitoxantrone [338] or for the co-delivery of PS and drugs. In this context, light-triggered release of small molecule multi-inhibitor — cabozantinib/XL184, in combination with nanoliposome-mediated PDT (PMIL; Photoactivatable multi-inhibitor nanoliposome), has been shown to induce cytotoxicity at extremely low doses of the multiple RTK pathway inhibitor cabozantinib/XL184 [41]. In this unique strategy, as demonstrated in Fig. 12, NIR irradiation, following intravenous administration of nanoconstructs not only led to photodamage of tumor cells and microvessels but also accelerated the release of the small molecule inhibitor (cabozantinib/XL184) in the tumors. The subsequent suppression of VEGFR and MET signaling by cabozantinib/XL184 led to a significant decrease in tumor volumes. Interestingly, the combined treatment not only inhibited tumor growth *in vivo* but more importantly there was a dramatic decrease in metastatic escape after a single treatment. The submicromolar doses of cabozantinib/XL184 (0.1–0.125 mg kg^−1^) used in this study did not induce cancer cell death as a single agent thereby reducing the risk associated with drug toxicity and dose interruptions. Although various promising studies have shown success in preclinical models, the role of nanomedicines in mechanism-based design of combination PDT-based therapies is yet to be exploited to the full extent.

**Fig. 13.**
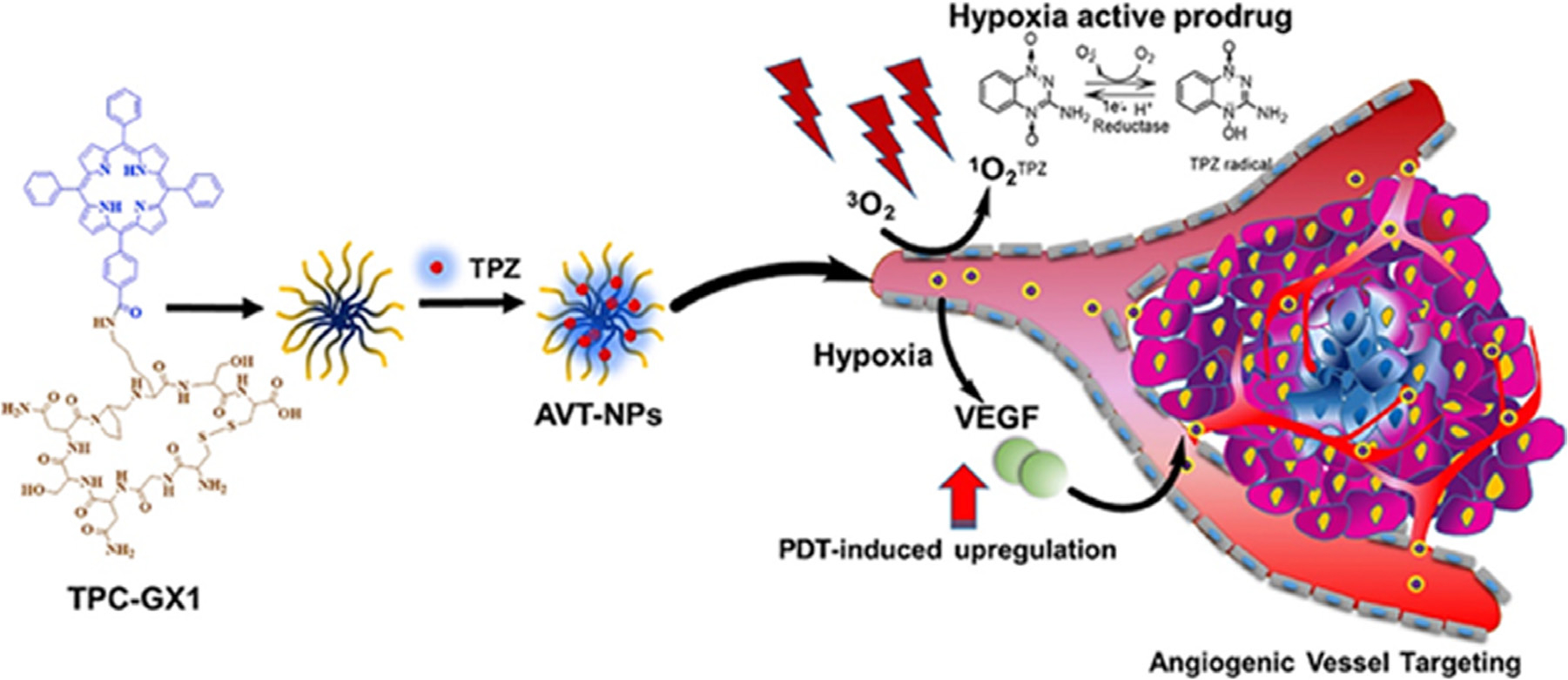
Schematic illustration of the formation of angiogenesis vessel-targeting NPs (AVT-NPs), generation of cytotoxic Tirapazamine (TPZ) radical under hypoxic conditions in cancer cells, and illustration of AVT-NP/TPZ based PDT that induces a local hypoxic environment and promotes angiogenesis for targeted drug delivery and synergistic chemo-phototherapy. Figure adapted from Guo *et al.* (2017) [342].

Combinatorial approaches, utilizing multicompartmental nanoconstructs carrying multiple payloads or conjugation of PSs to therapeutic carrier molecules (peptides, serum proteins, antibodies) for selective vascular or cellular damage [277, 282, 339, 340] can be designed for mechanistically informed therapy or to bring a synergistic effect that can lead to reduction in drug doses and systemic toxicity. Not only are the cell death mechanisms important but also the intracellular targets of the anticancer agents, as this will likely affect how the combination with PDT affects the tumor cells. Pharmacokinetics and tumor localization of encapsulated drugs ultimately enhance antitumor efficacy, as compared to unencapsulated drugs [341]. But, for successful combination therapy, the selection of a drug synergetic with PDT is crucially important, which depends on the cell death mechanisms of the chemotherapeutic drug in question. Guo *et al.* designed angiogenesis vessel targeting NPs (AVT-NPs) for chemo-photo synergistic cancer therapy (Fig. 13) [342]. The anti-cancer effect was achieved first by PDT, immediately followed by hypoxia-activated cytotoxic free radicals. With targeting capability, the AVT-NPs effectively accumulated at the tumor site due to angiogenesis, promoted as a response to PDT-induced hypoxia. The more nanoparticles delivered to the tumor tissue, the higher efficacy of PDT can be achieved, resulting in a more severe hypoxia and increased angiogenesis. Therefore, the prodrug embedded AVT-NP functions as a positive feedback amplifier in the combinatorial chemo-photo treatment and achieved an enhanced anti-tumor effect, both *in vitro* and *in vivo*.

**Fig. 14.**
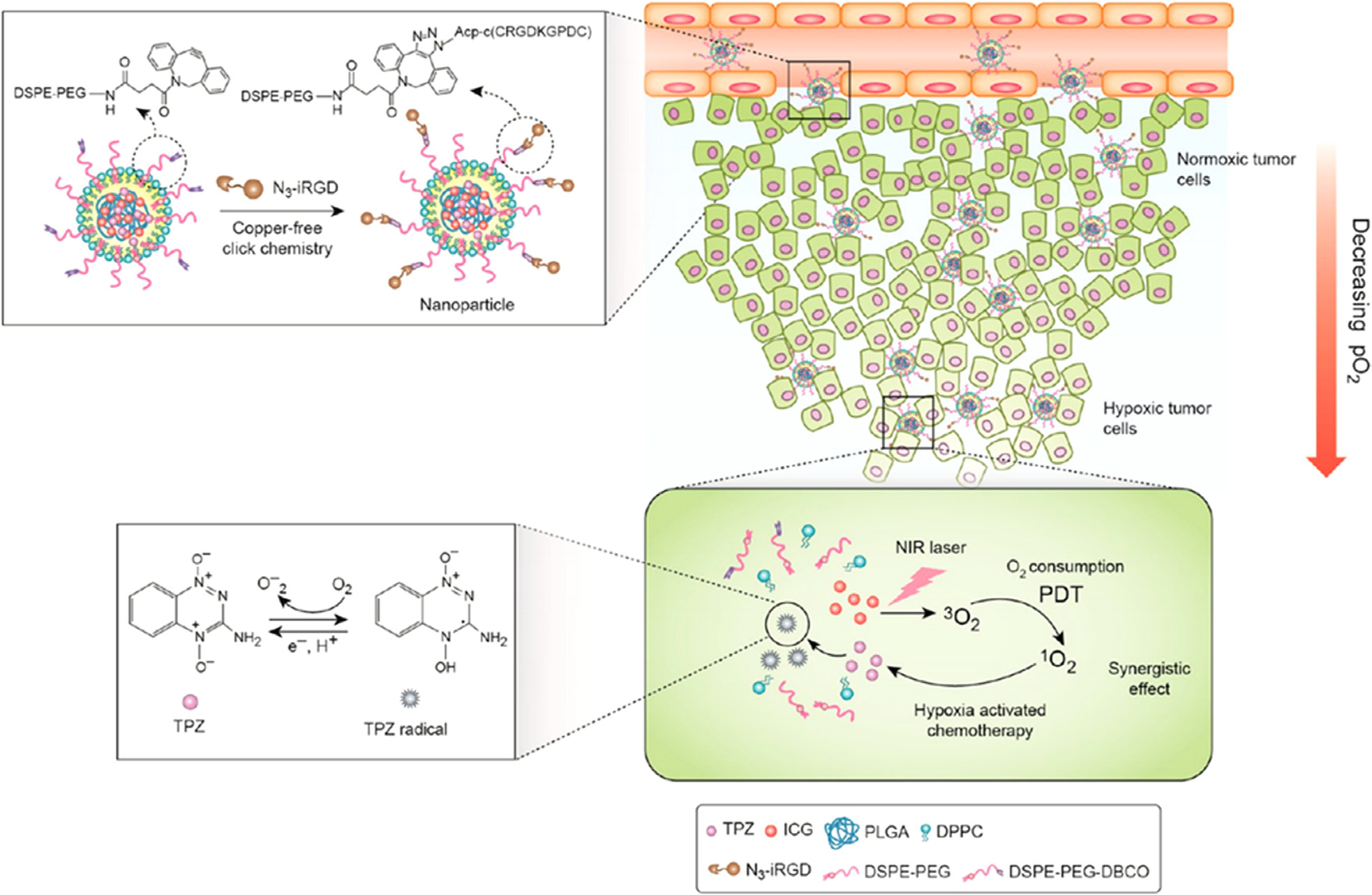
Mechanism of action of tumor-penetrating nanoparticles in a combined PDT and hypoxia-activated treatment strategy. Figure adapted from Wang *et al.* (2017), Copyright © 2017, American Chemical Society [346].

Cooperative interactions among various monotherapies may exert a characteristically synergistic/additive effect providing new ways for the efficient regression and even elimination of drug-resistant, hypoxic solid, or distant metastatic tumors. The emergence of hypoxia-activated prodrugs (AQ4N, TPZ, TH-302, PR-104A, NLCQ-1) [343, 344] provides new insight in cancer treatment. It may, however, exert unsatisfactory antitumor effects since tumors show hypoxic heterogeneity. Interestingly, PDT aggravates tumor hypoxia and promotes the activation of hypoxia-activated prodrugs [345]. The combination of PDT and hypoxia-activated prodrugs could suppress tumors. Wang *et al.* fabricated iRGD (a dual-targeting cyclic peptide; targeted to tumor vasculature and tumor cells) modified nanoparticles loaded with ICG and TPZ (Tirapazamine) for enhancing penetration and anti-tumor efficacy (Fig. 14) [346]. Due to active targeting by iRGD, the nanoparticles showed significantly increased penetration both *in vitro* and *in vivo.* Importantly, PDT could activate TPZ for synergistic anti-tumor effects because of tumor hypoxia amplification.

In contrast to PDT being used as an activator of prodrugs for enhancing their therapeutic potential, the combination of photothermal therapy with PDT has been used to enhance the therapeutic effects of PDT. Theoretically, the photothermal effect results in a local temperature increase [347–349], thereby speeding up blood flow to attract more oxygen for PDT. This could also be used to enhance the permeability of tissues and cell membranes to improve the delivery efficiency of nanoparticles and cellular uptake of photodynamic agents, thus forming the basis of PDT-based combinations with photo-thermal therapy. In this context combination of PDT and PTT has been shown to achieve a synergistic therapeutic effect to enhance treatment efficacy [350].

As mentioned earlier, an important attribute to the PD*P* process is the elicitation of immunogenicity in different tumors *via* the activation of ICD [20, 65, 170, 174]. Moreover, the recent success of immunotherapy, particularly targeting the immune checkpoint molecules (ICM) and rescuing the anti-tumor function of patient’s own T cells, has raised attention to the key role played by the immune system in the host defense against cancer [351]. ICM also termed as inhibitory receptors expressed on the surface of healthy immune cells are orchestrated to deliver tight immunological homeostasis in T cell-mediated immune responses in maintaining self-tolerance [352, 353]. Nevertheless, the expression of ICM leads to immune escape of tumor cells and some of these ICMs (particularly the cytotoxic T lymphocyte antigen (Ag)-4 (CTLA-4), programmed cell death-1 (PD-1) and its ligand (PD-L1)) are now being blocked by monoclonal antibodies (mAbs) [353–355]. Anti-CTLA-4, anti-PD-1 and anti-PD-L1 Abs were granted approval from the FDA and European Medicines Agency (EMA) for the treatment of a broad spectrum of neoplastic diseases [356], in early and advanced settings, because they were shown to elicit durable clinical responses. To date, however, clinical benefits from these treatments have been observed in only a subset of patients, likely, due to a lack of tumor immunogenicity.

PD*P* in combination with immunotherapy, thus, provides hope for enhancing anti-tumor effects as shown by reports from Wenbin Lin’s group and others [65, 179, 357]. PDT in combination with various ICMs has been explored in several preclinical models and such combinations have shown promise with long-term disease-free survival with effective tumor regression [65, 179, 180, 357–364]. A series of studies by Wenbin Lin’s group have shown that not only a local tumor regression but also systemic long-term anti-tumor immune responses could be generated by a combination of PDT and immunotherapy, that are capable of rejecting distant untreated tumors [179, 180, 357]. Combination approaches of PDT with ICM inhibitor-based immunotherapy are most effective when there is some temporal control for maximizing therapeutic potential [179]. In addition, several studies combining PDT with immuneadjuvants such as CpG oligodeoxynucleotides (for DC activation) [365] or small molecule metabolic checkpoint inhibitors such as 1-methy tryptophan [366], have also been reported. Nanotechnology-based approaches have been particularly advantageous in co-delivering these molecules with PS for spatiotemporal coordination. Although, the action sites of these immune adjuvants and metabolic inhibitors are in the TME, their effect on immune activation is realized at distant sites as well through the abscopal effect. All these observations hold much promise for PDT based immunotherapy and need further investigations for patient-specific personalization and its clinical use.

#### Actively targeted photonanosensitizers and photonanomedicine.

**Fig. 15.**
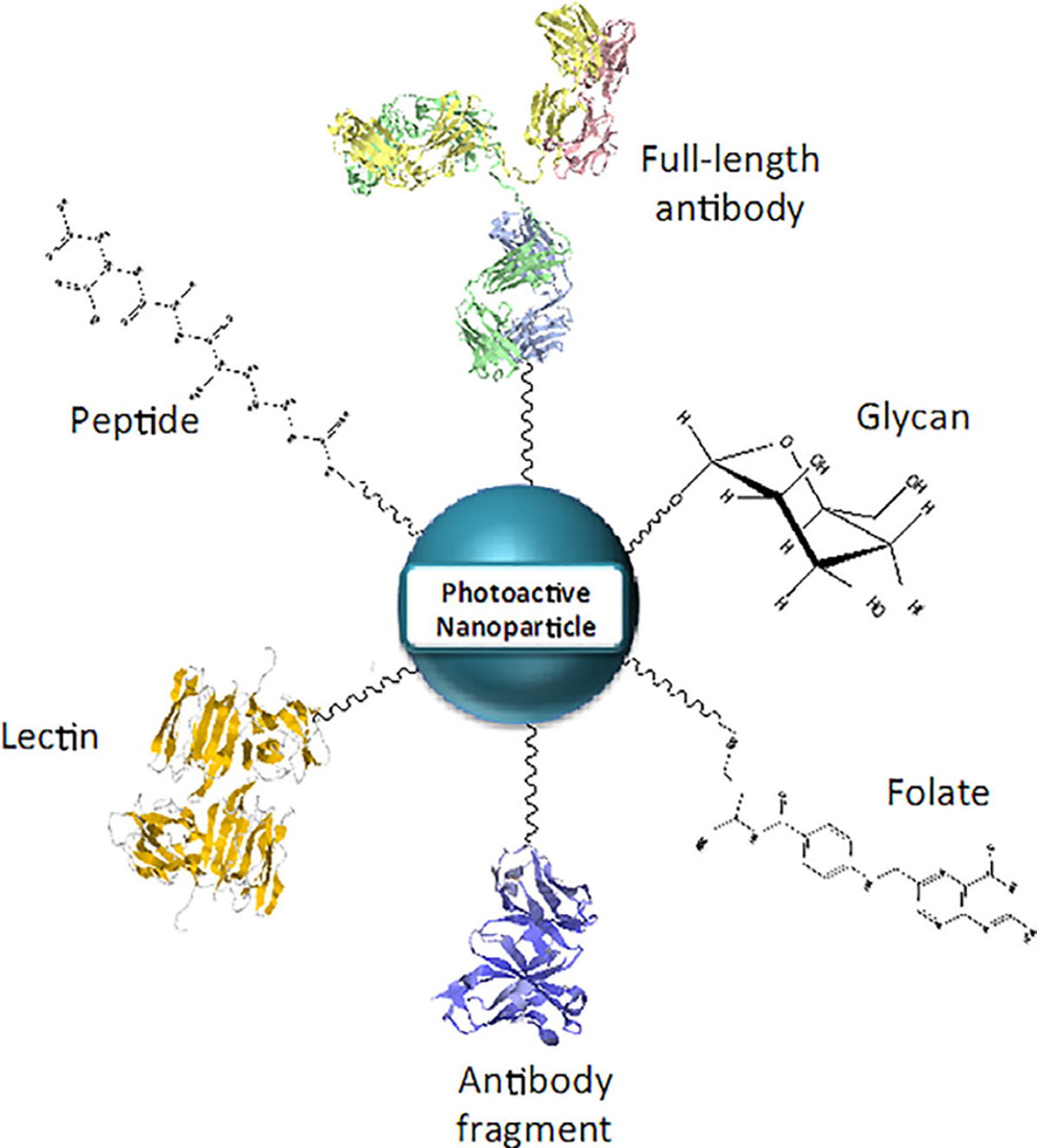
A schematic presentation of targeting ligands used to functionalize nanoconstructs to mediate the molecular selectivity of PDT damage. These include mAbs and their fragments, glycans, folate molecules targeting the folate receptor, and peptides. Figure adapted from Obaid *et al.* (2016), reproduced by permission of The Royal Society of Chemistry [280].

Active targeting intends to augment tumor cell-specific uptake of drugs and involves coupling of targeting moieties including folate, RGD peptides, full-length antibodies (mAbs), Fab’ fragments, peptides, small molecule ligands and aptamers (Fig. 15) [367] to the photosensitizers (drugs) or PS-formulations (nanoconstructs), enabling them to selectively bind to cancerous cells to increase effective drug concentrations [280, 284, 368].

Owing to their specificity for antigens, monoclonal antibody (mAb) mediated active targeting approaches have been the gold standard for over the past two decades. Moreover, as most of the preferred mAbs bind to overexpressed receptors on tumor cells, they also provide therapeutic benefits by suppressing signaling pathways involved in uncontrolled cellular proliferation and migration [369]. Many mAbs used in tumor targeting are FDA approved drugs (biologics) for tumor control and management [370]. Interestingly, BPD-PDT has been shown to synergize with anti-EGFR (Cet) therapy due to the targeting and inhibition of non-overlapping molecular pathways [371], thus forming the basis of PICs. These PICs target cell surface antigens, inducing highly selective cell death after NIR light exposure [372]. Savellano *et al*. conju gated verteporfin (BPD) to the anti-EGFR antibody; Cet, showing the targeting specificity of the conjug ate to EGFR expressing human cancer cells [373]. The conjugate promoted targeted photodamage of squamous cell carcinoma and ovarian adenocarcinoma cells, whereas free verteporfin exhibited no specificity. The exciting progress in PIC-mediated PDT (PIT) has led to the ongoing Phase II trials (NCT02422979 and NCT03769506) for head and neck cancers [374, 375], with initial results showing promising therapeutic potential. Photo-immunoconjugates, often, require a higher PS to antibody ratio in order to impart significant phototoxicity, however, overloading of PSs to mAb at times may lead to a loss of binding activity [197]. As an effective PIT-regimen requires a threshold concentration of intracellular PS, to enhance PS uptake yet retain antibody selectivity, Huang *et al.* demonstrated a nanoengineering approach of immobilizing PICs onto nanocarriers successfully directing their internalization to the target cells, and enhancing PS delivery [332].

Another molecular target that has been widely exploited for PDT is the human epidermal growth factor receptor 2 (HER2). Several studies with HER2 targeted PDT have established the efficacy of this target in enhancing selectivity in various tumor types including preclinical models of disseminated ovarian and gastric tumors [376, 377]. Anti-HER2 immunoliposomes were reported to selectively bind to and internalize in HER2-overexpressing cancer cells *in vitro,* and doxorubicin-loaded anti-HER2 immunoliposomes exhibited marked therapeutic effects in HER2-overexpressing xenograft models [378]. A wide variety of targeted gold, silica, polymeric and liposomal nanoparticles [379–382] functionalized with antibodies (anti-EGFR, anti-HER2), lectins [383], folate molecules [384], peptides (tripeptide Arg–Gly–Asp (RGD) [385, 386], cyclic version of RGD, CRGDKGPDC (called iRGD) [387]), aptamers [388] and mannose [389] have been designed for selective cancer cell uptake with enhanced PDT efficacies. In this context, combination therapies employing multiple targeting moieties either as a cocktail or conjugated to liposomes have also been studied, and shown to enhance selectivity and efficacy in PDT-based approaches [390, 391].

Apart from targeting moieties, nanoconstructs and their surfaces can be extensively modified to endow them with multiple functionalities, including modifications for increasing cellular internalization and accumulation at the target tissue, long systemic circulation or organelle-specific drug delivery [282], with the challenge being to strike a balance between toxicity and efficacy. Moreover, as the TME is heterogeneous, with several cellular and non-cellular components, exclusively targeting tumor cells in TMEs could be important. In an elegant study, Obaid *et al*. showed an efficient strategy for the photodestruction of desmoplasia in heterotypic pancreatic tumors with the molecular precision of antibody-based therapeutics. This study established a platform for the delivery of chemi cally tuned NIR activatable targeted photoimmuno-nanoconstructs (PINs), demonstrating an effective binding and penetration into the tumor, with respect to the non-targeted nanoconstructs leading to significant photomodulation of tumor collagen, which is considered to be a major barrier to drug delivery, drug distribution, and immune-cell infiltration in the TME [392–394].

While targeting surface markers has been extensively reported, targeting nuclear molecules has not been achieved with the same amount of success. The major barrier to this is the endosomal degradation of the targeting moiety and the nanoconstruct. Following the success of several anti-cancer drugs that have their targets in the nucleus, nanoagents targeted to nuclear molecules including DNA are also being considered [395, 396]. Intranuclear delivery of PpIX and subsequent *in situ* PDT in the nucleus damages nuclear DNA and induces apoptosis, enhancing PDT efficiencies both *in vitro* and *in vivo* [396]. Although nano-carriers transport the cargo to the tumor cells reasonably well, one of the barriers to achieve their anti-neoplastic effect is the endosomal escape to reach their target, which often impedes their action. PDT can be applied to facilitate cytosolic delivery of macromolecular drugs to their intracellular targets [397]. In this context, photochemical internalization (PCI) has made significant advancements. This approach features endocytic escape, as pioneered by Berg and colleagues [398], utilizing low/optimal doses of PSs and light, that are only sufficient for the selective rupturing of endocytic vesicles of the targeted cells, resulting in the cytosolic release of therapeutic contents. The strategy is being applied for many applications including gene delivery and clinical treatment of cancers where enhancement in chemotherapeutic drug delivery is needed [399].

**Fig. 16.**
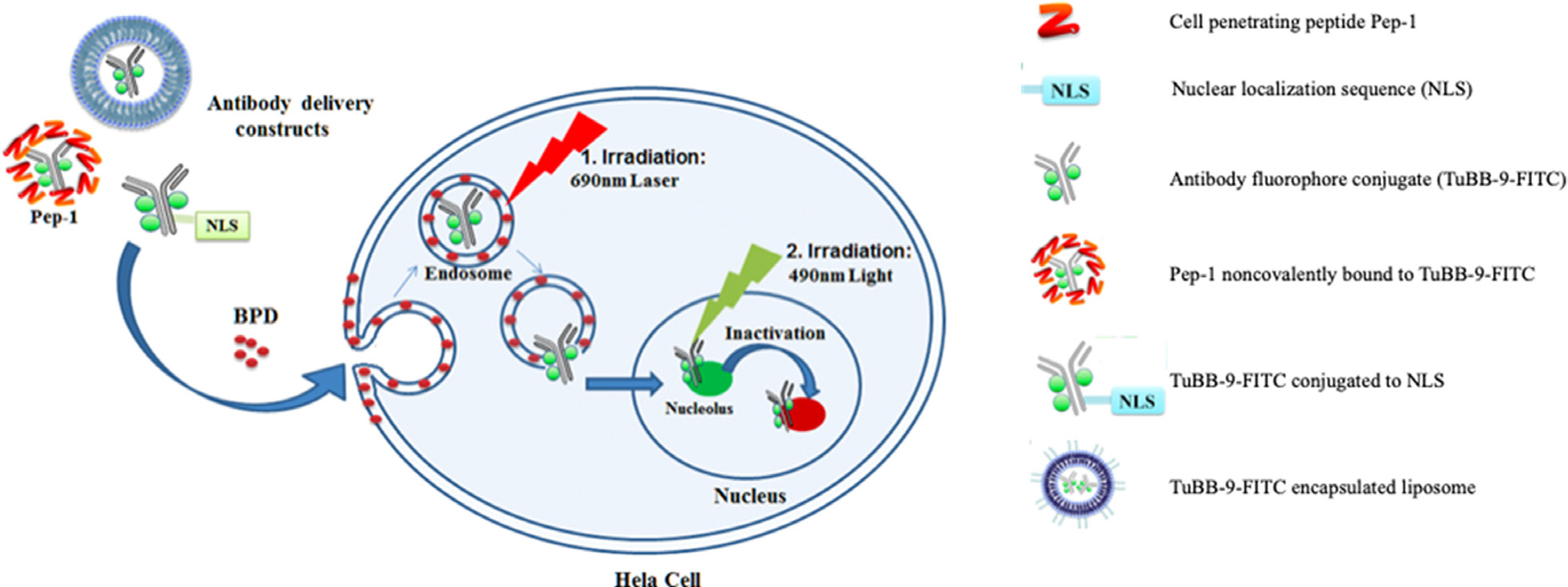
Light-controlled two-step approach for selective delivery of cell penetrating light activatable nanoconstructs for targeted photoinactivation of Ki-67 protein. Figure adapted from Wang *et al.* (2015), Copyright © 2015, American Chemical Society [402].

As the selective photodamage and inhibition of intracellular and nuclear proteins is a promising strategy to improve PDT efficiency Rahmanzadeh *et al*. demonstrated an exciting approach to target and photo-inactivate the nuclear proliferation protein Ki-67 [400]. PIC-encapsulating liposomes (PICEL) delivered PICs, targeted to Ki-67, intracellularly in a two-step irradiation approach. PICs carried by PICELs were released in the cytoplasm through photodynamic activation of BPD leading to endo-lysosomal rupture and release of intact PICs, which otherwise are degraded in the endolysosomal complex through protease action. PICs released in the cytoplasm translocate to the nucleus where subsequent irradiation of the PICs inactivated Ki-67 in a process commonly referred to as chromophore assisted light inactivation (CALI) [400, 401]. Based on the established ability of PICs to selectively cause substantial photodamage to the proliferating ovarian cancer cells, Wang *et al*. demonstrated the phototherapeutic cellular destruction by a two-step light triggering strategy [402]. This involved the administration of two PSs in two different formulations to induce 1) endosomal escape of the FITC conjugated Ki-67 antibody through the first irradiation step (690 nm for BPD) in a PDT treatment similar to PCI, and 2) Subsequent irradiation at 490 to inactivate the nuclear Ki-67 protein and induce cell death. The synergistic effect of irradiation with nuclear targeting led to efficient cell death with molecular selectivity (Fig. 16). Despite the existence of novel approaches employed for tumor-selective and targeted PDT, it is becoming increasingly clear that a “one size fits all” approach will not work [403]. However, advancements in nanomedicine and precision imaging techniques will potentially allow for personalized approaches geared for patient specific outcomes.

## PHOTODYNAMIC TREATMENT OF PATHOGENS: ANTIMICROBIAL PDT

Although this review primarily focuses on PDT of cancer, non-cancerous pathologies also benefit from the photodynamic processes. Of these, the best known is the treatment of AMD, mentioned earlier, which became a clinical and commercial success [404, 405]. Another promising area for PDT; referred to as antimicrobial PDT (*a*PDT), is briefly discussed below. In this section, selected reports and potential future applications of *a*PDT are summarized. The section by no means claims to be a comprehensive review of the field and for thorough discussions on *a*PDT, the reader is referred to other reviews [406–409] and, to somewhat dated but comprehensive papers by Hamblin and Hasan [1] and Wainwright [410, 411].

*a*PDT has been studied primarily for extracellular bacteria but there have been clinical studies against intracellular pathogens discussed later in the section. The emergence of drug-resistant pathogens and the agnosticism of *a*PDT to this resistance open up new opportunities for antimicrobial photomedicine [408, 412]. Photodynamic action against microbes follows the same light-triggered processes, as in mammalian cells, and resistance mechanisms similar to antibiotic resistance have not been reported, thus far [413]. This may primarily be because PDT is not applied repeatedly to allow for escape pathways to evolve. Given the dramatic increase in antibiotic resistance in the past years, a therapy that can be applied to a variety of strains and potentially against multi-drug resistant (MDR) strains could change the landscape of infection. PDT has shown promising activity against a broad range of pathogens, for example, clinically important bacteria including extracellular, Gram (+) (*Staphylococcus aureus*, *Enterococcus faecalis* and *Streptococcus pyogenes*) and Gram (−) (*Escherichia coli, Klebsiella pneumonia, Helicobacter pylori, Acinetobacter baumannii and Pseudomonas aeruginosa*) strains. The modality has also been successful against intracellular, Gram (+) (*Listeria monocytogenes*) and Gram (−) (*Salmonella Typhimurium*) bacteria as well as the acid-fast *Mycobacteria* species [414–418]. There have also been numerous reports for the success of PDT against parasitic infections such as leishmaniasis, trypanosomiasis, schistosomiasis, and malaria, to name a few. The methodology has also shown efficacy against pathogenic fungi (*e.g. Candida* species) and viruses (*e.g.* human papillomavirus) [419–424].

**Fig. 17.**
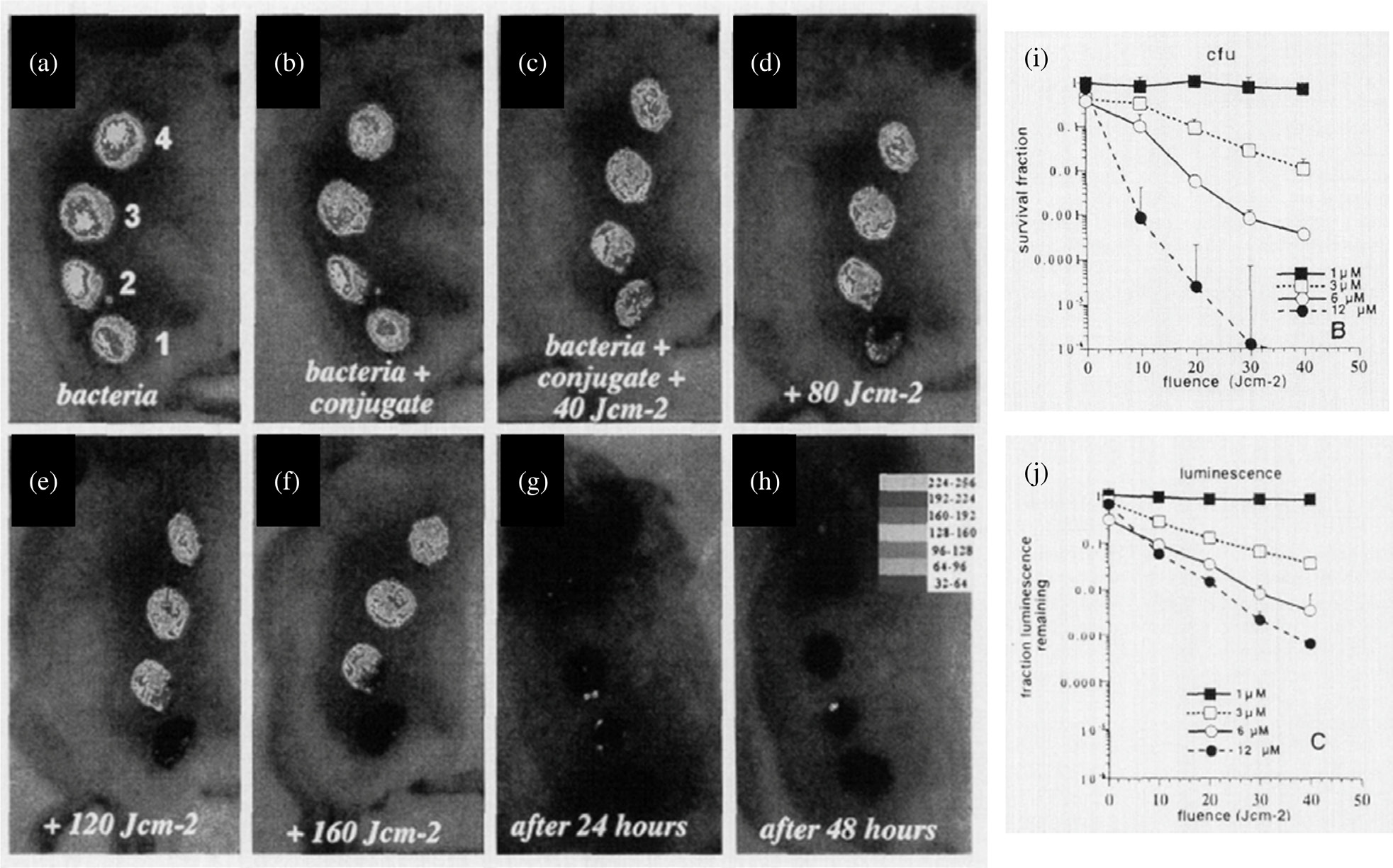
Mouse with wounds infected with E. coli, treated with conjugates (PS plus polymer) followed by illumination at 660–665 nm with different fluence rates along with phototoxicity analysis *in vitro*. (a) Four excisional wounds, (b) wound 1 and 2 received topical application of the conjugate, (c)–(f) wounds 1 and 2 were then illuminated with different fluence rates (40–160 J/cm^2^), (g), (h) the same mouse 24 h and 48 h later. Phototoxicity of the conjugates towards bacteria determined by (i) CFU counts, and (j) luminescence assays. Figure adapted from Hamblin *et al.* (2002) [434].

Some of the earliest studies of *a*PDT of bacteria reported killing of *E. coli* with neutral red [425] and acridine orange [426]. Studies, in the 1990s, on Gram (+) and Gram (−) bacteria led to the current understanding of susceptibility of bacteria towards PDT and importance of PS charge; with neutral or anionic charged PSs demonstrating the greatest *a*PDT effect against Gram (+) bacteria [427], due to differences in their cell wall [415, 428]. The cell wall of Gram (+) bacteria allows PSs to easily pass through due to the thick porous layers made up of peptidoglycan and teichoic acid [427]. The teichoic acid on the outer membrane contributes to impart an overall negative charge to the bacteria which serves as an additional attractive force for cationic PSs [429]. Contrary to this, Gram (−) bacteria have a thin peptidoglycan layer close to the inner cytoplasmic membrane, an outer membrane made of phospholipids and a negatively charged lipopolysaccharide. As a result, this creates a permeability barrier which limits the attachment of anionic and lipophilic PSs to the bacteria [429]. Studies by the group led by Nitzan have followed traditional strategies of overcoming the permeability problem by using permeability enhancers [427, 430, 431], whereas the group led by Wainwright repurposed the use of PSs with intrinsic (+) charge, such as toluidine blue [432], to transport PSs across the Gram (−) outer membrane for greatest *a*PDT efficacy. Different conjugation approaches have also been applied in an effort to enhance the antimicrobial effects of PDT. For example, in a study, the anionic PS (chlorin e6) was conjugated with cationic-charge-bearing polymers which showed successful increase in PS-conjugate penetration through the outer membrane of Gram (−) bacteria as shown in Fig. 17 [433, 434]. More interesting findings came out from the group led by Jori using cationic porphyrins (*e.g.* TMPyP) [435], where they concluded that washing the loosely bound PS on the outer surface of bacteria reduced the photodynamic effect. On the other hand, if the PS was not washed, following illumination, the permeability of the PS was enhanced due to initial damage, which resulted in greater killing [435]. Until the late 1990s, many studies had shown *in vitro* anti-bacterial efficacy of PDT but there were only a few studies that demonstrated selective killing of pathogens compared to host cells [436]. To this end, a study done by Zeina *et al.* in 2001, to kill skin microorganisms (including *Streptococcus pyrogens, Staphylococcus aureus and Staphylococcus epidermidis*) using PDT, was one of the initial investigations that showed selective damage of the bacteria compared to the host keratinocytes. Currently, a wide variety of other PSs such as methylene blue [437], rose bengal [438], porphyrins [439] and indocyanine green [440] have shown promise in *in vitro* studies of *a*PDT. More recently, the relatively old concept of dual-action antibiotics [441] such as tetracyclines (which act as antibiotics in the dark and photosensitizers with light activation) has received some attention [42, 442–444] and warrants further investigation.

Other than success with *in vitro* and *in vivo* models of bacterial infection, *a*PDT has been explored as a potential clinical treatment in randomized clinical trials, with most studies targeting periodontal disease [445–447]. In 2015, a clinical trial performed by Alwaeli *et al.* (Jordan University of Science and Technology), involving a conventional treatment (scaling and root planning) for periodontal disease combined with *a*PDT suggested significant improvement in all evaluated clinical parameters for at least 1-year post-treatment. An interesting application to note, although not yet studied in clinical trials, is a 2018 publication by the Hamblin group, in which methylene blue (MB) and potassium iodide (KI) mediated *a*PDT was utilized to treat urinary tract infections (UTIs) in a rat model [332]. Drug-resistant UTIs are painful, difficult-to-treat and often require repeated antimicrobial therapy. The authors utilize the well-known PS, MB combined with KI to potentiate *a*PDT, followed by delivery of intravesicular illumination with a diffusing fiber connected to a 660 nm laser. Significantly shorter infection duration was found in the *a*PDT treated rats compared to controls, and a smart solution to monitor treatment efficacy was shown *via* bioluminescent imaging of the bacteria following treatment up to 6 days post-*a*PDT. Further work needs to be performed for full translation into clinic, but light-based methods for the treatment of antibiotic-resistant bacteria is an exciting new approach with potentially simple clinical adaptations. In particular, *a*PDT against biofilm growth, which is characterized by unique capabilities to thwart traditional antibiotic treatments and cause recurrent infection, will likely continue to develop [406, 408, 448].

**Fig. 18.**
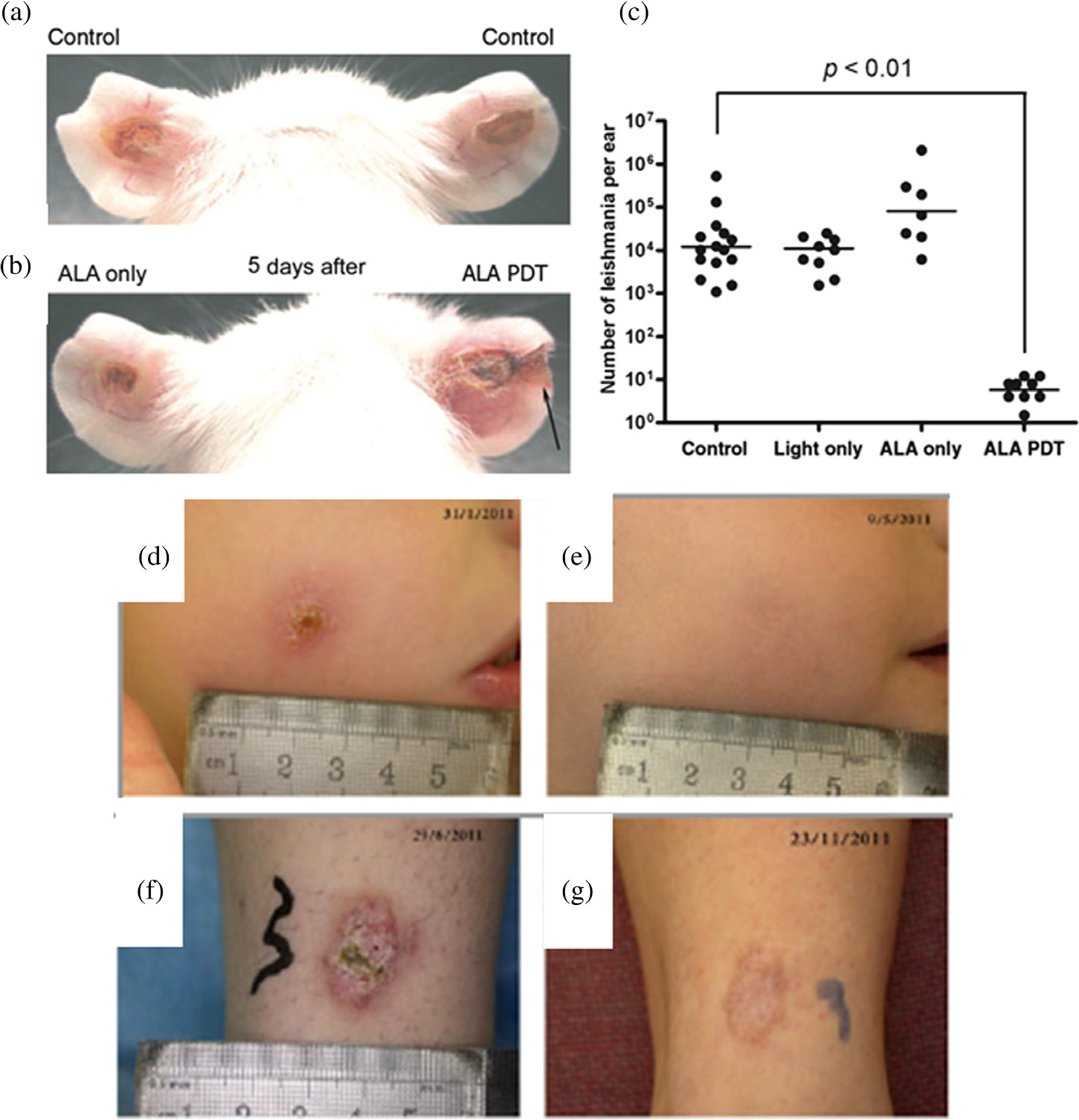
Efficacy of PDT against *Leishmania* in preclinical models. (a) Control ears with no treatment, (b) Topical ALA-PDT on cutaneous leishmaniasis (CL), (c) Quantification of *L. major* parasites. (d)–(g) Clinical outcome of patients with cutaneous leishmaniasis before (d) and (f) and after treatment (e and g) with daylight-activated photodynamic therapy (DA-PDT). Figure adapted from Akilov *et al.* (2007) and Enk *et al.* (2015) [459, 462].

Intracellular pathogens have also been shown as reasonable targets for PDT. The mechanisms are naturally complex as the PS now has to traverse the cell to find organelles harboring the pathogens. Brovko *et al.* showed bactericidal efficacy of PDT, *in vitro,* against intracellular pathogens including *Salmonella* and *Listeria* using a range of PSs (Rose Bengal, acriflavine neutral, phloxine B, and malachite green) [449]. Other major intracellular pathogens studied for *a*PDT, include *Mycobacterium spp.*, Cutaneous Leishmaniasis (CL), and malarial parasites. An interesting approach has been aimed to control vectors that act as host reservoirs to complete different parts of the parasite life cycle *e.g.* mosquitoes, snails, and flies. Abdel-Kader and coworkers used naturally derived porphyric PSs such as chlorophyll and hematoporphyrin derivates as photopesticides [450–452]. They used sunlight to activate the PS to control these noxious insects which act as host reservoirs for these pests. However, the methodology had limited scope due to human safety and environmental concerns. Currently, pathogen directed approaches are being researched to control parasites, leveraging photodynamic mechanisms. Among all the parasites, *Leishmania* is the most studied pathogen in the field of antimicrobial photomedicine. PDT has shown excellent results against this otherwise tedious organism and is worth discussing. *Leishmania* parasites are engulfed by the macrophages and proliferate inside them while escaping the host immune response by producing reducing agents against oxidative stress [453]. Generally, PDT can neutralize these effects by overexpression of cytokines, such as IFN-γ, eliciting an immune response to kill intracellular parasites [424, 454]. Even though PDT has shown great potential to reduce *Leishmania* parasitic load, as shown in preclinical and clinical studies (Fig. 18), there are varying reports in terms of the mechanism of eradication. For example, *L. amazonensis* parasites are resistant to IFN-γ mediated killing [455]. On the other hand, in the case of *L. major*, IFN-γ signaling in infected macrophages promote overexpression of nitric oxide (NO) and nitric oxide synthase (iNOS, NOS_2_), which together with RMS generated during phagocytosis, kill the parasites [456, 457]. In one clinical study [458], ALA-PpIX based PDT showed a complete absence of lesions of *L. major*, 28 days post-PDT, with absence of amastigotes. However, other studies [459, 460] indicate that even though PDT shows favorable results, the parasites were not completely eliminated. Furthermore, since the authors did not find significant differences in the expression levels of different cytokines in PDT treated versus non-treated parasites, they concluded that PDT-mediated killing of the parasite was primarily indirect. Contrary to this, Souza *et al.* report an increase in IFN-γ in response to ALA-PDT against *L. braziliensis* and conclude that the reduction in parasite load was through both direct and indirect mechanisms [461]. However, much work needs to be done before we can generalize the mechanism of action of PDT against *Leishmania* which may vary among different species. Recently, a clinical study in Israel done by self-administering topical methyl aminolevulinate followed by daylight PDT has reported much success to treat cutaneous leishmaniasis [462], which due to ease can be adopted in developing countries. MB has also been successfully used to treat leishmaniasis [463].

Among all viral infections antiviral PDT has been most successful against human papilloma virus manifestations. Another area that has earned much success over the past years for photodynamic inactivation of viruses is decontamination of blood products [464]. Of particular importance for blood disinfection is the use of different PSs such as riboflavin (vitamin B2) [465], methylene blue [466], and psoralen (a derivative of amotosalen) [467]. PDT has also been successful in inactivating viruses in plants, food products, water, and environment contaminants. To this end, curcumin [468], hypericin [469] and porphyrin [470] are only a few from the long list of PSs used to inactivate different types of viruses. With the efficacy of different antiviral photodynamic applications, aPDT’s ability to bypass known resistance mechanisms makes it a promising tool against viral infections [465].

### Exploiting defense mechanisms of bacteria for targeted PDT

**Fig. 19.**
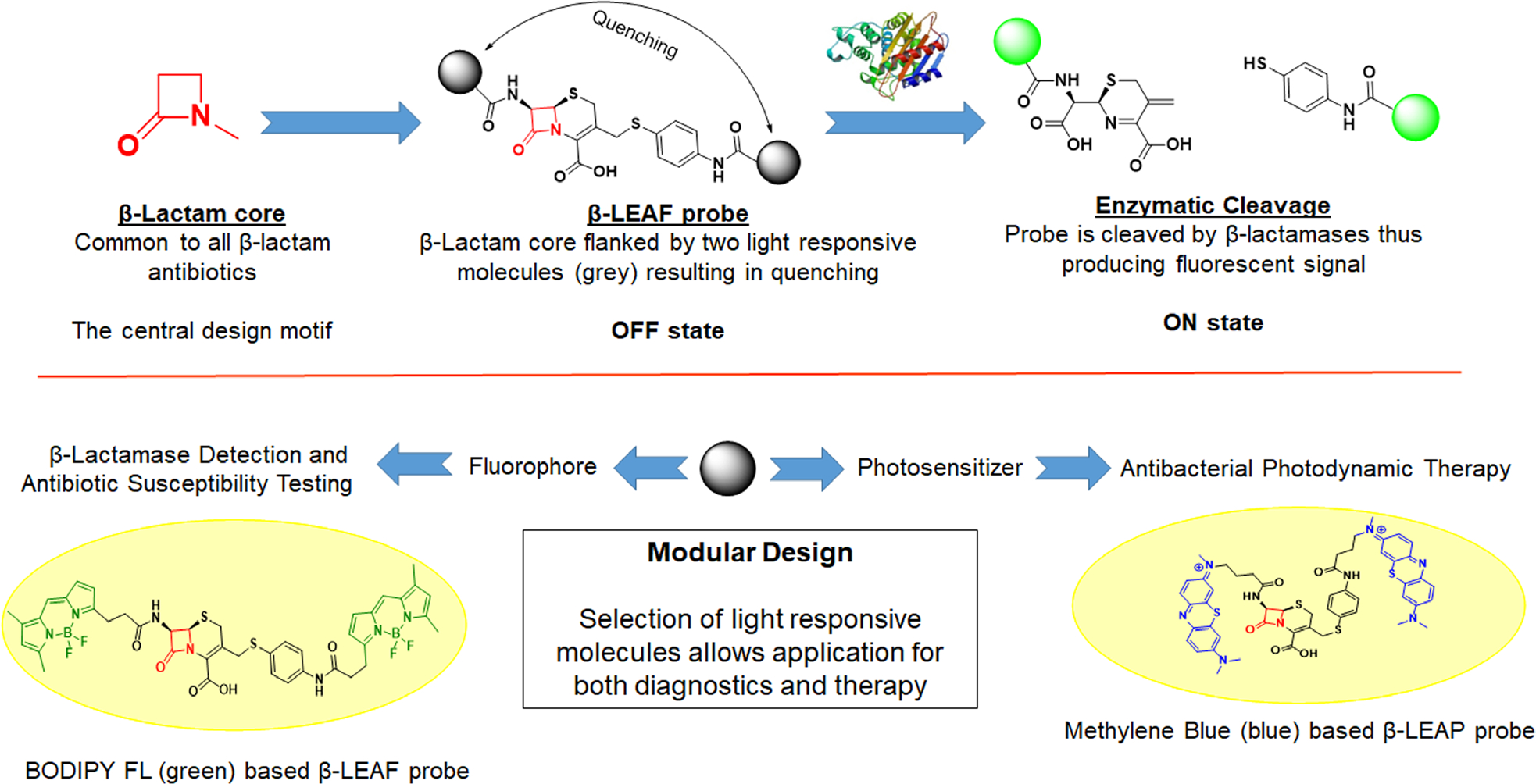
Schematic representation of the β-LEAF and β-LEAP strategies.

β-lactamase is an enzyme that is produced by the bacteria as a resistance mechanism against β-lactam antibiotics and is absent in mammalian cells. One special concept exploiting this bacterial defense mechanism is based on the phenomenon of quenching and dequenching of the attached PS or fluorophore. The name of the approach and consequently the probe is β-lactamase activated photosensitizer (β-LEAP) or β-lactamase activated fluorophore (β-LEAF) and can be used for treatment and diagnosis respectively. The design of the system and probe contains a cephalosporin core including the β-lactamase cleavable lactam ring conjugated to two chromophore moieties [416, 471–476]. When the probe is intact the PS or fluorophore is quenched and upon cleavage is dequenched [477]. Over the years, β-LEAP/β-LEAF technology has excelled and has gone through much development. As a result, the methodology is being investigated with different PSs (*e.g.* methylene blue) and fluorophores (*e.g.* BODIPY FL) (Fig. 19). This approach is one of the examples by which PDT can achieve a more selective and specific therapeutic effect even against resistant bacteria. Moreover, the concept can be used to exploit other resistance mechanisms and turn them against the bacteria. On the other hand, β-LEAF can be used for the detection of β-lactamase production and prediction of antibiotic susceptibility, a two-pronged strategy. This technique has an advantage in antibiotic susceptibility testing regime where rapid diagnostic testing is necessary for informed decision making. The modality from a diagnostic standpoint is now being used for carbapenemase detection and typing to predict antibiotic resistance within 10 min. The strategy is comparable and gives superior results *vs*. traditional approaches which can take up to 24–48 h [478]. The approach is also successful for photodynamic inhibition of different carbapenemases which in turn restores susceptibility to the previously resistant drugs [479]. Contrary to the traditional antibiotic susceptibility testing regime another inherent advantage of β-LEAF is its capacity of being a functional assay (real-time predictors of antibiotic resistance). The technology can also be opted against other organisms, for instance, against filarial parasites, as most filaria have an endosymbiont called *Wolbachia*, which is essential for the survival of the parasite and is often β-lactamase positive. Thus, the β-LEAF/β-LEAP platform can be utilized for efficient diagnosis and treatment in filaria management programs.

### Exploiting host–parasite symbiosis for targeted PDT

**Fig. 20.**
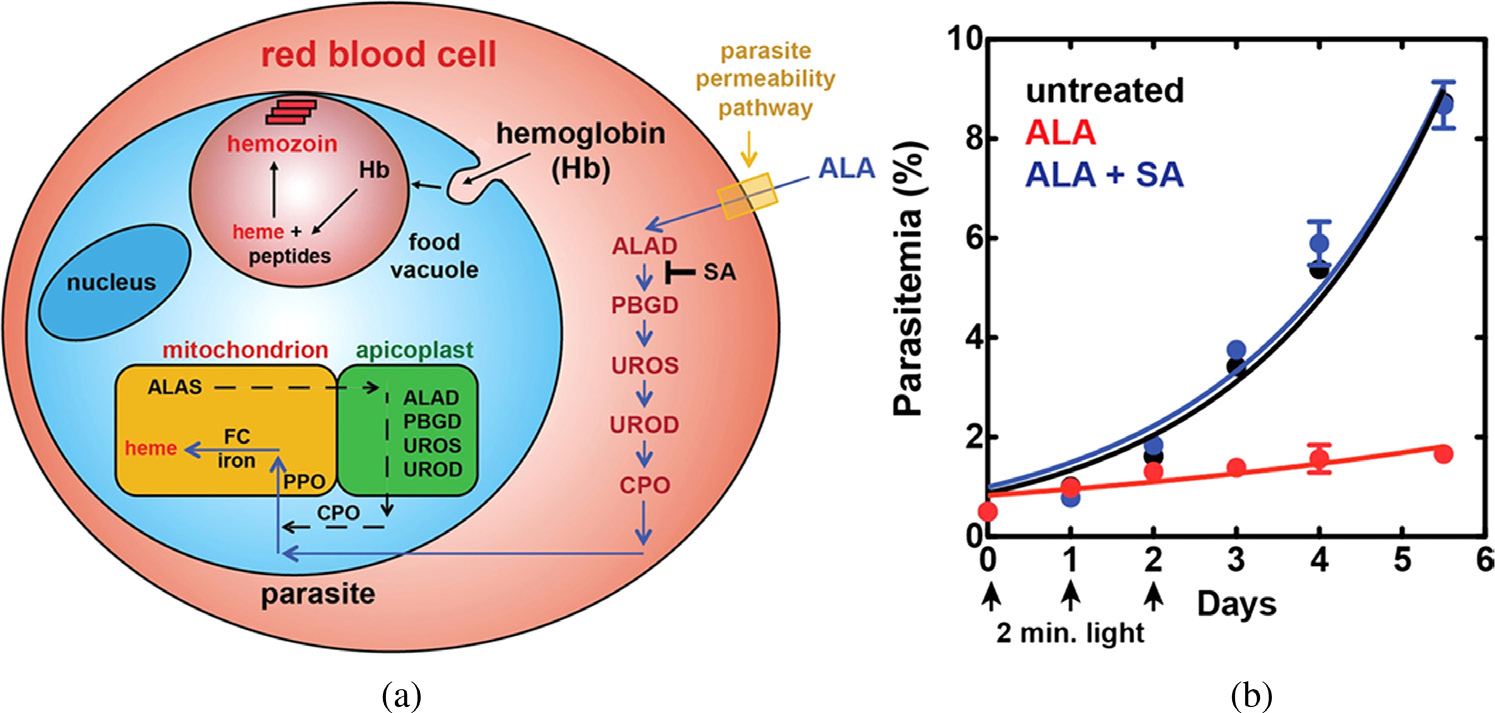
Schematic diagram and results of ALA-PDT against malaria (a) Depiction of ALA-uptake and porphyrin biosynthesis pathways in Plasmodium-infected erythrocytes. (b) Growth of malaria parasites in the presence or absence of ALA and succinyl acetone (SA, an agent used to block PpIX synthesis), with light exposure. Figure adapted from Sigala *et al.* (2015) [480].

Like other organisms, parasites also deploy unique host–pathogen interactions for their survival. More recently, exploiting such mechanisms, Sigala *et al.* have shown selective targeting of *Plasmodium falciparum*, the causative organism of malaria [480]. The authors utilized the heme synthesis pathway by incubating the pro-photosensitizer, ALA, with *Plasmodium*. Interestingly, when red blood cells (RBC) get infected by parasites, they hijack the machinery of RBCs and develop new nutrient acquisition pathways. As a result, this new permeability machinery enhances selective uptake of ALA into the infected RBCs as compared to the uninfected RBCs. Feeding parasites with exogenous ALA results in an increase in the production of parasite’s PpIX, one of the precursors of heme. In this process, the infected RBCs become photoreactive and upon irradiation generate RMS which in turn kills the parasites, as shown in Fig. 20. Even though this study has significant drawbacks and could be improved by using a specific wavelength of light (which in turn would allow reducing the concentration of ALA), it opens up new avenues for targeting heme synthesis machinery by PDT. For instance, often external (*e.g.* ticks and mites) and internal (*e.g.* babesia and theileria) parasites have unique nutrient acquisition pathways that can be manipulated by leveraging photodynamic mechanisms.

### Potential of anti-microbial PDT applications

Even though PDT has a long-standing history of clinical use mainly in cancer, the phenomenon of *a*PDT has also provided many options for the treatment of infectious diseases. The main advantage of *a*PDT is that it is effective against both drug-susceptible and resistant pathogens [481, 482]. Unlike antibiotics, which target a specific pathway of an organism, PDT kills or inhibits the pathogen by a relatively nonspecific mechanism. This might be the primary reason that even after repeated exposures to the process, convincing evidence for the development of resistance has not been reported thus far [483–487]. Moreover, compared to other anti-infective agents, the same PS can be used against a variety of organisms [432]. In addition to this, *a*PDT has the ability to alter lipopolysaccharides, (a potent immune stimulant) which can induce secretion of pro-inflammatory cytokines by host cells [488–490]. Thus, the ability of PDT to not only kill or inactivate the organism but also act as an immunomodulatory process, gives it a dual advantage. The modality can also be used as a standa-lone therapy or in conjunction with other anti-infective agents. Moreover, it can also be used as a priming mechanism for enhancing the efficacy of different drugs and has the potential to reduce selection pressure and restore or reverse the efficacy of novel antibiotics [479]. PDT has also shown tremendous progress through targeted antimicrobial applications by exploiting known biological mechanisms and can contribute in making real-time decisions [414, 476, 480].

## PERSPECTIVE

The essential principles of PDT have been used in therapy since the days of the early Egyptians over 5000 years ago and have been developed significantly in recent years. Much has become known about mechanisms involved and the complexity of the PDT process so that the modality can now be investigated not only as a local disease control approach but also as a systemic therapeutic option in combinatorial regimens. The process of being subjected to the PDT transforms not only the cells targeted with light but also the microenvironment, both at the cellular and tissue level. The changes are often transient, *e.g.* the upregulation of VEGF secretion or increase in tumor permeability [491–496], and can be captured for increased responsiveness with complementary treatments. This phenomenon of transformation of tumor components transiently is referred to as PD*P*, as it primes the tumor for secondary complementary treatments to enhance the therapeutic outcome and deter any unwanted side-effects such as increased metastasis observed with almost all treatments [42, 497]. PD*P* may be viewed as an enabler of other therapies and forms the rationale for PDT-based combinations, where one treatment mechanism reinforces the next. Both PDT and complementary treatments may be administered separately [42] or simultaneously in nanoconstructs [41]. PDT based systemic immune effects are also a result of a PD*P* effect [179, 357, 498]. Although, PDT has limitations in its own, (such as depth of light penetration), recently there have been advances in the use of nanotechnology-based PDT, increased ICD due to PD*P* and combinatorial treatments in preclinical studies showing promising outcomes even at sites remote to the locally illuminated ones. So, one might look forward to a day when PDT is more than a local therapy using a deliberative, mechanism-based understanding and exploitation of PD*P*. The same is true for targeted treatments that provide a higher level of selectivity than is possible with small molecules [198, 499]. This allows for illumination of larger areas of the body, even those containing vital organs, such as in the abdominal cavity, without danger of irreparable collateral toxicity [198]. The ongoing Phase II trials (NCT02422979 and NCT03769506) with photo-immunotherapy, even though focused on local disease, is exciting and the community looks forward to the results of these trials [374, 375]. The other area of PDT that is upcoming is the use of the property of most PSs not only to elicit photochemistry but also to have finite quantum yields of fluorescence. This has led to the development and FDA approval of image-guided surgery [203, 294, 500, 501] with a PDT agent and opens up the option, in principle, of treatment following surgery in the same procedure [201]. PD*P* enhanced immuno-oncology is a future focus in the field. PDT mediated destruction of tumor cells happens primarily *via* necrosis and other cellular death mechanisms including apoptosis. While inducing cell damage or death, PDT releases DAMPs, which activate the innate and adaptive immune system components and enhance ICD. Local inflammation due to PDT-mediated recruitment of immune cells to the site of irradiation and increase in the infiltration of cytotoxic T lymphocytes can kill the tumor cells. Thus, the photodynamic process primes the TME, which may be beneficial in treating less immunogenic “cold” tumors and turning them into more inflamed “hot” ones compared to traditional treatments such as chemotherapy which can be immunosuppressive. Approaches for the use of PDT to treat non-cancerous diseases are also moving although, since the initial excitement and the success of the treatment of AMD, there have not been significant clinical approvals where PDT is used as a first-line treatment. From the end of 2019, with the COVID-19 pandemic, viral decontamination in patients and/or in devices such as ventilator inlets using PDT could prove to be an extremely useful technology. Preclinical and clinical studies suggest that *a*PDT, along with nanotechnology and photochemically primed immune system and combination treatments are likely to be the next areas of advanced development/application of PDT.
